# Investigation of Radiosensitivity Gene Signatures in Cancer Cell Lines

**DOI:** 10.1371/journal.pone.0086329

**Published:** 2014-01-22

**Authors:** John S. Hall, Rohan Iype, Joana Senra, Janet Taylor, Lucile Armenoult, Kenneth Oguejiofor, Yaoyong Li, Ian Stratford, Peter L. Stern, Mark J. O’Connor, Crispin J. Miller, Catharine M. L. West

**Affiliations:** 1 Translational Radiobiology Group, The University of Manchester, Manchester, United Kingdom; 2 Experimental Oncology Group, The University of Manchester, Manchester, United Kingdom; 3 Gray Institute for Radiation Oncology and Biology, The University of Oxford, Oxford, United Kingdom; 4 Applied Computational Biology and Bioinformatics Group, CRUK Manchester Institute, Manchester, United Kingdom; 5 Immunology Group. CRUK Manchester Institute, Manchester, United Kingdom; 6 Cancer Bioscience, Astra Zeneca, Macclesfield, United Kingdom; Ludwig-Maximilians University, Germany

## Abstract

Intrinsic radiosensitivity is an important factor underlying radiotherapy response, but there is no method for its routine assessment in human tumours. Gene signatures are currently being derived and some were previously generated by expression profiling the NCI-60 cell line panel. It was hypothesised that focusing on more homogeneous tumour types would be a better approach. Two cell line cohorts were used derived from cervix [n = 16] and head and neck [n = 11] cancers. Radiosensitivity was measured as surviving fraction following irradiation with 2 Gy (SF2) by clonogenic assay. Differential gene expression between radiosensitive and radioresistant cell lines (SF2</> median) was investigated using Affymetrix GeneChip Exon 1.0ST (cervix) or U133A Plus2 (head and neck) arrays. There were differences within cell line cohorts relating to tissue of origin reflected by expression of the stratified epithelial marker p63. Of 138 genes identified as being associated with SF2, only 2 (1.4%) were congruent between the cervix and head and neck carcinoma cell lines (MGST1 and TFPI), and these did not partition the published NCI-60 cell lines based on SF2. There was variable success in applying three published radiosensitivity signatures to our cohorts. One gene signature, originally trained on the NCI-60 cell lines, did partially separate sensitive and resistant cell lines in all three cell line datasets. The findings do not confirm our hypothesis but suggest that a common transcriptional signature can reflect the radiosensitivity of tumours of heterogeneous origins.

## Introduction

Intrinsic radiosensitivity is an important factor underlying radiotherapy response [1]. Radiosensitivity can be measured as the fraction of cells surviving a single 2 Gy dose of radiation (SF2) with high values indicating radioresistance. While other methods are available to measure cellular radiosensitivity in cell lines, SF2 is considered to be the gold standard and is supported by strong clinical evidence. *In vitro* measurements of SF2 correlate with *in vivo* radioresponse in mouse models [2]. Measurement of SF2 in primary human tumours was an independent prognostic factor in patients with carcinoma of the cervix [3] and head and neck [4] following potentially curative radiotherapy. Despite the evidence for its importance, no method is available for its routine assessment in patients, due to the impracticalities of measuring tumour radiosensitivity. The ability to measure a tumour’s radiosensitivity would be a major advance and allow individualised treatment to reduce dose and/or omit chemotherapy in patients with sensitive tumours or conversely to intensify treatment against resistant tumours. Treatment individualisation should increase survival and reduce morbidity. Estimates suggest a biologically individualised approach to treatment based on radiosensitivity testing could increase survival rates by >10% [5].

Consequently there is interest in deriving a gene signature that reflects radiosensitivity. Several methods have been explored: identifying genes induced following irradiation in cell lines [6]; identifying differential expression between induced radioresistant and parental radiosensitive cancer cell lines [7] and profiling the *in vitro* response of cervix tumours to irradiation [8]. Most published studies were small and have not been independently validated. The most comprehensive studies used the NCI-60 panel of cell lines [9]. One study identified 22 genes that together discriminated between low and high SF2 values in 63 cell lines, based on a threshold of 0.2 (i.e. cell lines with less than 20% colony survival following 2 Gy defined as radiosensitive) [10]. Another series of studies developed a predictive classifier of radiosensitivity based on SF2 associated gene expression profiles in the NCI-60 lines [11], [12], [13], [14]. The endpoint of these studies was a regression model of 10-hub genes, which had prognostic significance when applied to three clinical datasets (rectal, oesophageal and head & neck cancers) [13] and was also predictive of benefit from radiotherapy in breast cancer [15]. Additionally a meta-analysis of published data from four microarray platforms for NCI-60 cells identified a 31 gene radiosensitivity signature [16].

The NCI-60 panel is the most extensively characterised set of cancer cell lines and a public resource that is frequently used as a screening tool for drug discovery [9]. The panel contains cell lines from multiple tissues of origin but few radiobiologically relevant tumour types such as cervix (n = 0) or head and neck (n = 0), i.e., cancers where radiotherapy is an important part of treatment. It is well known that tumours derived from different tissues vary in radiosensitivities; with haematological malignancies being sensitive, and glioblastoma and melanomas the most radioresistant [17]. Studies show that basal gene expression levels correlate strongly with tissue of origin, particularly between haematological and solid tumours [10]. As such, considerable variation and noise is present in the NCI-60 ‘basal’ gene expression data, potentially hampering the identification of genes associated with SF2. The transcription factor P63 is a marker of squamous cell origin and regulates many genes associated with epidermoid/squamous cell fate. Loss of p63 is associated with the up-regulation of genes associated with a more mesenchymal/migratory cell fate [18].

It was hypothesised that deriving a radiosensitivity signature using a more homogeneous group of cell lines would be a better approach. We obtained 16 cervical carcinoma cell lines, a tumour type where radiotherapy is important but that is not represented in the NCI-60 panel. The cells were characterised in tightly controlled basal conditions; parameters measured included SF2, protein expression by reverse-phase protein array (ZeptoMARK) and gene expression by Affymetrix Exon 1.0ST array. We attempted to identify genes that were differentially expressed between high and low SF2 cell lines in a single homogeneous tumour type. We had access to a second independent radiobiologically-relevant head and neck squamous cell carcinoma (HNSCC) cell line cohort (n = 11) to validate our findings and those derived from the publically available NCI-60 data.

## Materials and Methods

### Cell Lines

Fourteen commercially available cervical carcinoma cell lines were obtained from the American Type Culture Collection (ATCC) or the Japanese Collection of Research Bioresources (JCRB). Two other cell lines (778 and 808) were derived in house [19]. All cervix cell lines were cultured in identical conditions: 4.5 g/l glucose DMEM plus Glutamax (Life Technologies, Paisley, UK), supplemented with 10% foetal calf serum (FCS) (Lot: A04305-0160, PAA Laboratories (Yeovil, UK)) and kept in a humidified incubator. Eleven head and neck cell lines were cultured as described in Table S1. All cell lines underwent STR authentication and were mycoplasma free.

### Clonogenic Assays

The method is described elsewhere [20]. Briefly, exponentially growing cells were trypsinised and irradiated with 0–10 Gy at room temperature using an X-ray unit at a dose-rate of 1.37 Gy/min. Following plating and 2–3 weeks growth, the colonies formed were stained with crystal violet and those with >50 cells scored. Each experiment involved a minimum of three but usually six technical replicates and experiments were repeated two (n = 4) or three (n = 21) times. Data shown are the mean of the biological replicates.

### HPV Genotyping

The HPV genotyping of these cervical carcinoma cell lines was described previously [21]. For head and neck carcinoma cell lines qRT-PCR for E2, E6 and E7 for HPV16 and HPV18 was performed as described previously [22].

### MTT Assay

Doubling time was estimated for each cell line using the CellTiter 96 Aqueous Non-radioactive cell proliferation assay (Promega, Madison, WI, USA) as per manufacturer’s ‘overnight’ protocol. A standard 7-day growth curve was performed in 96-well plates. Colorimetric readings were taken at 570 nm and compared, by exponential regression to a standard curve of known cell density. An average of three independent replicates at different densities was used to calculate the mean doubling time.

### RNA Extraction

Cells were washed in PBS and snap-frozen in liquid nitrogen. RNA was extracted and DNase treated using the Qiagen RNeasy Kit (Qiagen, UK), as per manufacturer’s instructions. RNA integrity (RIN) and quantification were measured using a Bioanalyser (Agilent Technologies Ltd, Santa Clara, CA, USA). 260/230 and 260/280 ratios were assessed using a Nanodrop 1000 Spectrophotometer (Thermo Scientific, Wilmington, DE, USA).

### Western Blotting

The p63 protein status of the cervix carcinoma cell lines was described previously [21]. Using the same methods Western blotting was performed on the head and neck cell lines, using the following antibodies: p63 mouse monoclonal (BC4A4) (Abcam, Cambridge, UK) and anti-β-Actin mouse monoclonal (Clone AC-15) (Sigma-Aldrich, Dorset, UK).

### ZeptoMARK Reverse-phase Protein Arrays

Exponentially growing cells were washed with PBS, lysed in 75 µl of CLB1 lysis buffer (Zeptosens: a Division of Bayer (Schweiz) AG, Switzerland), scraped into microfuge tubes, vortexed and incubated at room temperature for 30 minutes. Samples were centrifuged at 15,000 rpm at room temperature, supernatants collected and concentrations determined by Bradford assay. The spotting procedure has been described before [23]. Briefly, cervix carcinoma protein lysates were standardised to 2 mg/ml, from which four concentrations (0.20, 0.15, 0.10 and 0.05 mg/ml) were spotted, in duplicate onto a ZeptoMARK hydrophobic chip (Zeptosens). Each cell line was independently grown and harvested on two occasions; consequently two biological replicates were spotted onto the array. Chips were blocked with CeLyA buffer (Zeptosens), before incubation with primary antibodies for 22 hours at 20°C. Twenty-four antibodies (Zeptosens) were selected based on their role in cancer or therapy resistance [24]. After incubation excess primary antibody was removed and a fluorescently-labelled species-specific antibody hybridised for 2.5 hours at 20°C. After washing, arrays were read on a ZeptoREADER (λ_ex_/λ_em_ = 635/670 nm). The resulting relative fluorescent intensity (RFI) was calculated from a standard curve constructed from the four concentrations (in duplicate). This is a quantitative protein measurement. Values displayed are the mean of two biological replicates (i.e. 4 standard curves).

### Exon Array Hybridisation

100 ng RNA was amplified using NuGen WT-Ovation FFPE v2 kit (NuGen Technologies, San Carlos, CA, USA). The WT-Ovation Exon Module V1.0 was used to generate ST-cDNA and 4 µg was hybridised to Human Exon 1.0 ST arrays (Affymetrix, Santa Clara, CA). Further details and raw data (CEL files) are available at http://bioinformatics.picr.man.ac.uk/vice (or GEO: GSE39066 (part of super series GSE39067). Raw data for HNSCC cell lines are available at GEO: GSE51370.

### Exon Array Data Analysis

Microarray data were normalised using RMA [25]. The R/BioConductor package *annmap* and the annmap database [26] were used to remove non-exonic and multi-targeting probesets. Array performance was measured as the percentage of probesets flagged as “present” with a conservative cut-off (%Detection Above BackGround [%DABG] *P*<0.01) and only those probesets flagged “present” in at least three samples were retained. This filtering reduced the number of probesets considered from 1,411,399 to 353,981 exonic probesets, of which 243,301 passed DABG filtering. Gene level summaries were calculated by taking the median signal of filtered probesets that mapped to unique gene symbols. When summarised this resulted in 31,345 genes considered. Unsupervised hierarchical clustering was performed on the 1000 most variant genes (ranked on coefficient of variation) to show the separation of samples based on the most variable genes in the data, while minimising computational requirements. Signature Generation: A gene signature was determined to be the set of genes or probesets that were significantly differentially expressed between two groups of cell lines according to either LIMMA or Rank Product Analysis. The cut-off for significance was a false discovery corrected p value of 0.01. Packages: R: 3.0.2, Annmap: v1.2.1 using human database build 66, LIMMA: 3.17.26, RankProd: 2.32.0, Pheatmap: 0.7.7.

### Validation Cohorts, Array Mapping and Data Analysis

Head and neck cell line Affymetrix U133A Plus2 array data were RMA normalised using the *affy* package in R. Affymetrix control probesets (‘AFFX’ annotated) were removed. For variance analysis, _x_, _a_ and _s_ annotated probesets were also removed. NCI-60 - Affymetrix Plus2 cel files were downloaded from CellMiner (http://discover.nci.nih.gov/cellminer/) and RMA normalised as before. After normalisation, replicate arrays for each cell line were averaged. For comparison to the gene-level summarised exon array data, Plus2 probesets were mapped to gene symbols using *annmap*.

### Radiosensitivity Signature Mapping

All signatures were applied to the gene-level summaries of the cervix data using gene symbol mapping. For application of signatures to the HNSCC and NCI-60 Affymetrix Plus 2 datasets, the following protocols were used:

1. Probeset IDs for the Eschrich *et al* [13] ten hub genes were taken from Table 3 from the group’s first paper [13]. NCI-60 test set cell lines were taken from Table 4 from the group’s second paper [12]. Twelve cell lines were listed but there was no corresponding Plus2 array for the breast cell line MDN.
2. The top four ranking genes from Torres-Roca *et al* [14] (*RPIA, RBBP4, RGS19, ZNF208*) were mapped to Affymetrix Plus2 probesets using *annmap*. The corresponding expression data for the probesets were extracted and plotted on a linear scale (anti-log).
3. Gene symbols for the Amundson *et al* gene signature were taken from the second table of the original article [10]. One gene could not be mapped (Unigene ID Hs.494347) as there was no corresponding gene symbol in the table. The remaining 21 gene symbols were mapped to Plus2 probesets using *annmap*. Multi-mapping probesets were removed.
4. The Tewari *et al* signature was taken from the second table of the original article [8]. Forty-nine of the 60 probesets with a unique gene symbol were extracted and mapped to Plus2 probesets using annmap. Multi-mapping probesets were removed.

Unsupervised analyses (clustering, PCA) of gene expression data, signature analysis and differential expression analysis (*LIMMA* [27], *RankProd*) were carried out using R. The threshold for differential expression using Rank Product Analysis (*RankProd*) was a Percent False Positive (PFP) rate of <0.01.

### Graphing and Statistics

Results show the mean of biological replicates and precision measurements are the standard error of mean unless otherwise stated. R values indicate Pearson’s product moment coefficient. Boxplots were generated in GraphPad Prism (v6.0): box-whisker parameters: horizontal bar indicates median expression, the box indicates interquartile range; whiskers represent the range. For visualisation of radiation survival curves a linear quadratic equation was fitted in R, with radiobiological parameters derived from DRFIT [28]. The R package *LIMMA*, was used to calculate differential expression values for protein profiling data. Where appropriate, *p*-values are Benjamini and Hochberg false-discovery rate (FDR) corrected [29]. Principal component analysis (PCA) reduces multi-dimensional data (i.e. thousands of genes) into data-points in 2-D space. The closer two data-points (samples) the more similar the samples. PC1 (x-axis) accounts for the majority of variance in an experiment, PC2 (y-axis) accounts for the component representing the second highest variance.

## Results

### Cervical Carcinoma Cell Lines have a Range of Radiosensitivities

**Figure 1:**
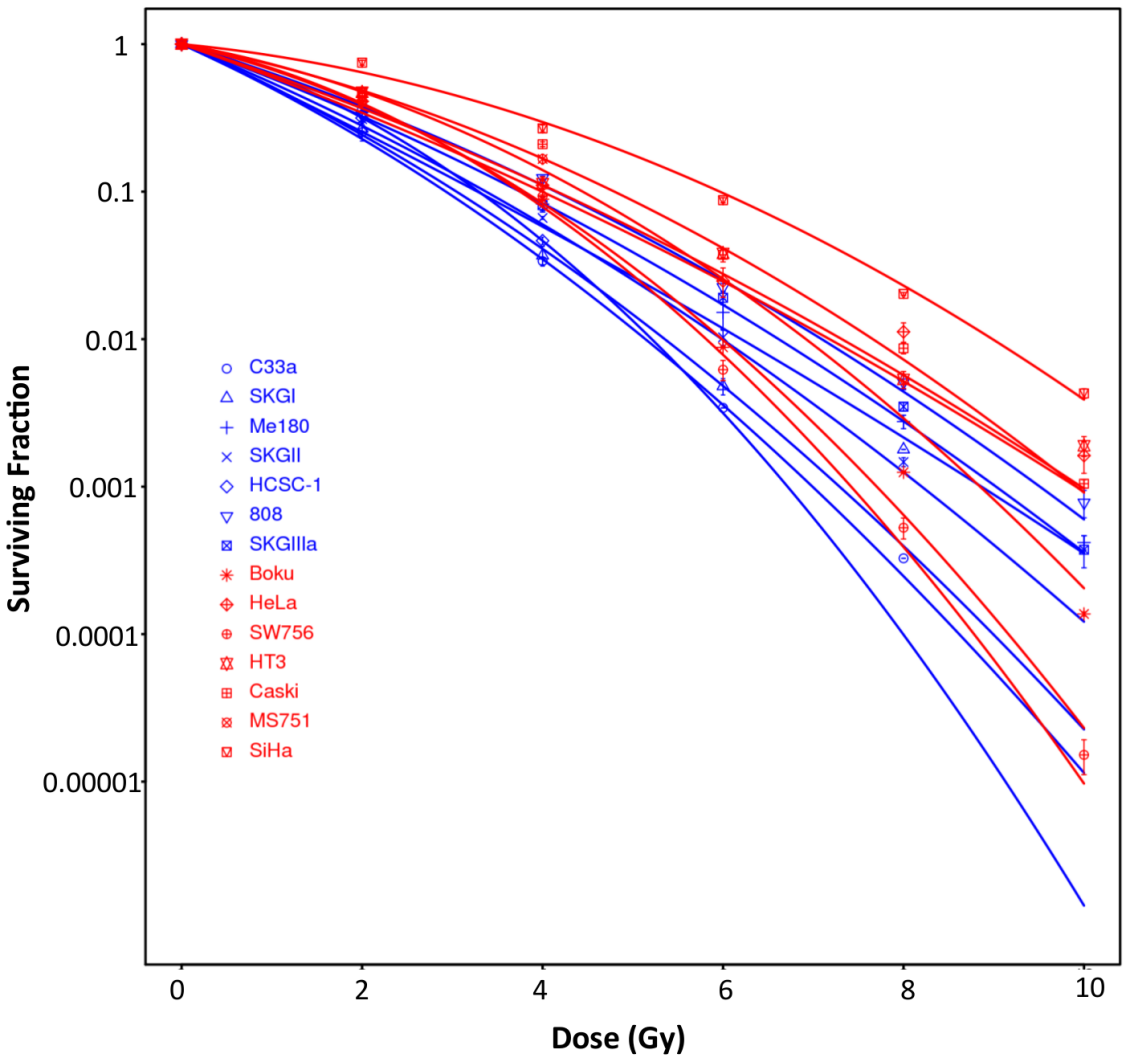
Radiobiological characterisation of cervix carcinoma cell lines. **A)** Radiation survival curves showing surviving fraction (log10) (y-axis) following irradiation with 2, 4, 6, 8 and 10 Gy for 14 cervix cancer cell lines. Data points are the mean and standard error of 2–3 independent experiments (3–6 replicates per experiment). Data-points are fitted with the linear quadratic equation and coloured by below (blue) or above (red) the median SF2.

Table 1 summarises the cervical carcinoma cell lines. Two cell lines did not form colonies and SF2 values for the remaining 14 lines ranged from 0.25 to 0.75 (Figure 1). SF2 values for six of the cell lines were published by another group [30], and the ranking was identical in both studies. In the 14 cell lines, there was no correlation of SF2 with plating efficiency (R^2^ = 0.005, *p* = 0.82), doubling time (R^2^<0.0001, *p* = 0.99) or the RNA expression of TP63, a marker of squamous cell differentiation (*p* = 0.90).

**Table 1:** Summary characteristics of the cervix cell lines.

Cell line*	Tumour histotype*	SF2	HPV Genotype$	p63† (Western)	TP63† (array)
Boku	SCC	0.41±0.01	16	–	4.24
C33a	Unknown	0.25±0.01	–	–	4.81
CaSki	SCC	0.47±0.02	16	+	8.67
HCS2	SCC	N/A	18	+	8.86
HCSC1	Small cell carcinoma	0.32±0.00	18	–	3.93
HeLa	Adenocarcinoma	0.41±0.04	18	–	4.54
HT3	Unknown	0.35±0.12	–	+	8.02
Me180	SCC	0.35±0.01	68	+	10.33
MS751	SCC	0.47±0.01	–	+	8.51
SiHa	SCC	0.75±0.06	16	–	4.93
SKGI	SCC	0.27±0.09	18	+	7.2
SKGII	SCC	0.31±0.02	18	–	5.65
SKGIIIa	Unknown	0.37±0.03	16	+	8.6
SW756	SCC	0.42±0.01	18	–	4.56
778	Unknown	N/A	18	–	5.19
808	SCC	0.33±0.02	18	+	8.28

* Provenance information from ATCC, JCRB or [17].

$ HPV genotype from [20].

† p63 expression from Western analysis and Exon derived array expression values from [19].

### Molecular Characterisation of Seemingly Homogeneous Cervical Carcinoma Cell Lines Shows Significant Disparity

**Figure 2:**
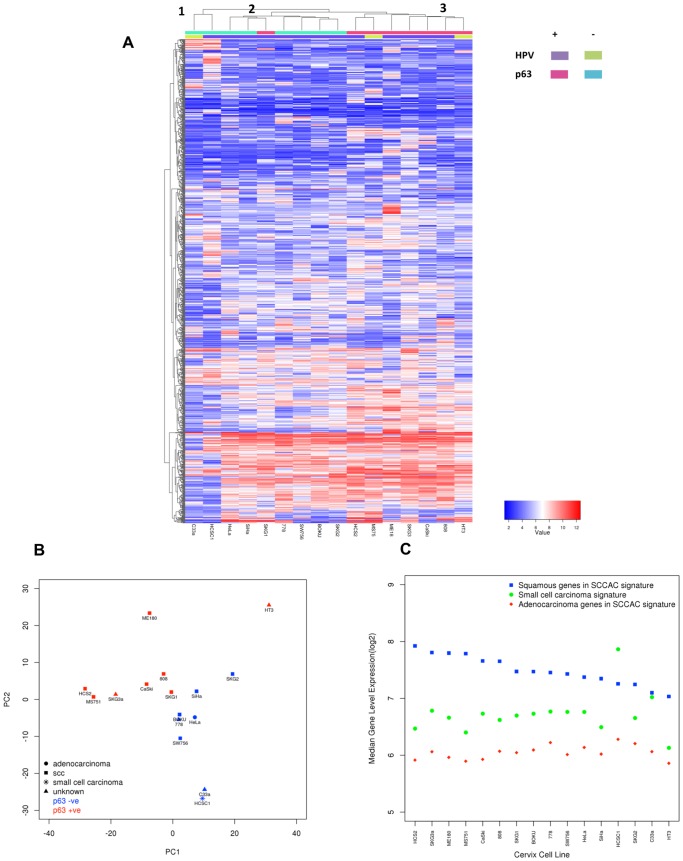
Transcriptional characterisation of the cervix cancer cell lines. **A)** Unsupervised hierarchical clustering of the top 1000 genes ranked by coefficient of variation (from Exon array data). Heatmap colouring is by log_2_ expression value. Rows represent genes and columns are cell lines. x-axis dendrogram (clusters) indicates the similarity of the cell lines and y-axis dendrogram the similarity of genes. Cluster 1 represents two samples with the lowest TP63 values (p63 negative). Cluster 2 shows the grouping of the other p63− cell lines including the adenocarcinoma HeLa. Cluster 3 groups p63+ cell lines, with the exception of SKG1, which is classified with p63 negative cells. **B)** Principal component analysis of the 16 cervix cancer cell lines based on SCC (n = 1062), AC (n = 155) and small cell carcinoma (n = 77) gene expression. The x-axis shows principal component 1 (PC1) accounting for 15.5% of the variance. PC2 displayed on the y-axis accounts for 13.7% of the variance in the histology signature gene expression. Colouring represents p63 protein expression. **C)** Graph showing the average expression (log2) of the SCC, AC and small cell carcinoma signature. y-axis is the Exon array derived median gene level expression, for each of three signatures. X-axis shows the cell line. Cell lines are ranked based on TP63 expression.

p63 expression (protein and mRNA) was measured because it discriminates between squamous (p63+) and non-squamous (p63−) histological types of cervix cancer [21]. Following transcriptional profiling, unsupervised clustering of the most variant 1,000 genes (ranked by coefficient of variation) separated the lines into three clusters (Figure 2A) with cluster 1 (C33a and HCSC1) being outliers. The other 14 cell lines partitioned as p63− and p63+ clusters with the exception of SKG1 which had the lowest TP63 transcript level of the p63 positive lines. HCS2 and 778, which did not form colonies in our conditions, did not cluster together suggesting no common transcriptional expression associated with ability to form colonies. These results suggest that the major basal transcriptional differences between the cell lines relate to p63 expression. Interestingly, while HeLa cells were the only adenocarcinoma (AC) according to provenance information, several cervix cell lines had similar global transcriptional profiles. HCSC1 is ‘small cell carcinoma’ derived, consequently we explored whether the clustering of C33a and HCSC1 was due to a shared histological origin. Principal component analysis (PCA) using the combined gene expression from two gene signatures, trained on (i) AC and SCC [21] and (ii) small cell carcinoma [31], showed that HCSC1 and C33a had very similar histological gene expression (Figure 2B). Figure 2C shows that C33a and HCSC1 had low levels of SCC genes and higher than average levels of small cell carcinoma genes. It is interesting to note that the AC gene expression was low in all cell lines, including HeLa, suggesting that this signature, derived in primary tumour material may have limited applicability in cell lines. These data suggest that C33a is histologically a small cell carcinoma derived cell line and highlights the transcriptional differences associated with histological type found in a relatively homogeneous single tissue of origin cohort.

### Protein Profiling of ‘Cancer Associated Genes’ shows Key Pathway Differences between Cell Lines, but not between High and Low SF2 Groups

**Figure 3:**
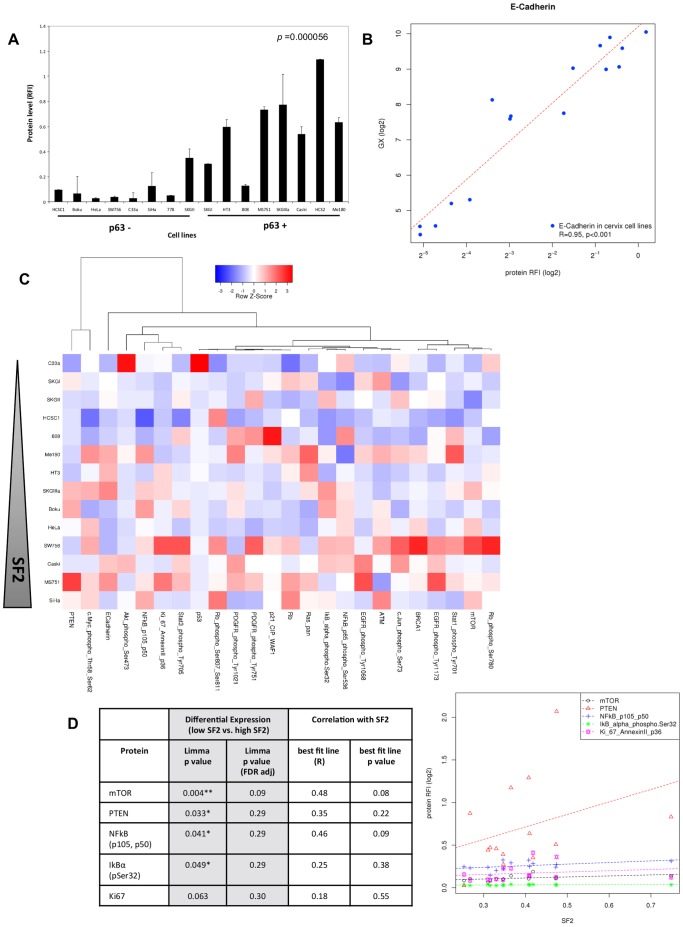
ZeptoMARK protein profiling of the cervix cancer cell lines. **A)** Histogram displaying the ZeptoMARK protein-array derived abundance for the 16 cervix cancer cell lines. The y-axis displays E-cadherin protein level (relative fluorescent intensity (RFI) for each of the cell lines (x-axis). Cell lines are ranked based on TP63 expression. Grouping into p63 negative and p63 positive cell lines confirms the association of E-cadherin with p63. The p value is T-test derived comparing the difference in E-cadherin expression between the p63 positive and negative groups, error bars display standard deviation of two biological replicates. **B)** x–y scatterplot showing E-cadherin gene expression (Exon array) on the y-axis against E-cadherin protein expression on the x-axis. Dashed line represents perfect correlation. Exon array data-points represent the average of multiple exonic probesets (n = 19) from a single Exon expression array, where protein data are the mean of two biological replicates. **C)** Heatmap showing clustering of proteins with similar expression (y-axis) in the ZeptoMARK protein profiling data. Cell lines ranked by SF2. Heatmap colouring is based on row Z-score. **D)** xy-scatter plot showing the expression (y-axis) of the top 5 proteins from LIMMA against SF2 (x-axis). Table summarises the results of Limma differential protein expression analysis between high and low SF2 groups and Pearson correlation of protein expression (RFI) against SF2. p values denote those proteins with differential expression (* p<0.05 or ** p<0.01) between SF2 low and high groups according to LIMMA analysis. However these fail to pass false discovery rate correction.

A panel of 24 proteins were selected from a catalogue of pre-validated antibodies of proteins implicated in cancer, or resistance to therapy [24]. Few DNA damage response antibodies were available and so selection was limited to well-validated proteins associated with cancer, such as p53, Rb, EGFR etc. As p63 is essential for the proliferative potential of stem cells in stratified epithelia [32], we postulated that p63+ cells would express higher levels of the epithelial marker protein E-cadherin, compared with p63− cells and this was confirmed by the protein array (*p* = <0.0001) (Figure 3A). We also compared the mRNA expression level of E-cadherin (Exon-array derived) with the protein abundance measured by the array (relative fluorescence intensity [RFI]; Figure 3B). There was a strong correlation (R = 0.95, *p*<0.001) demonstrating that protein levels reflect transcript levels for E-cadherin. We also detected high levels of p53 protein in C33a cells compared with all other cell lines (Figure 3C), due to a known mutation in the *TP53* gene [33] resulting in protein stabilisation. These data gave us high confidence in the protein profiling data. Unsupervised clustering of the protein data showed no relationships with known characteristics (Figure S1). Ranking the cell lines by SF2 showed no clear visual structure to the data (Figure 3C). The 14 cell lines were split into high and low radiosensitivity groups using the median SF2 value, as previously used with clinical specimens [3], [4]. Four proteins were differentially expressed (*p*<0.05) between the two groups: mTOR, PTEN, IκB alpha, and NFκB, but none were significant after false discovery rate (FDR) correction (Figure 3D, Table S2). mTOR was borderline significant (FDR *p = *0.09) and there was a trend for a moderate correlation between mTOR and SF2 (R = 0.48, *p* = 0.08, Figure 3D). These data reveal that while there were considerable differences between the cells in terms of protein expression and pathway activation, none of the proteins/pathways were robustly associated with SF2 in this cell line cohort.

### Head and Neck Cancer Cell Lines Show Similarities in Global Gene Expression

**Figure 4:**
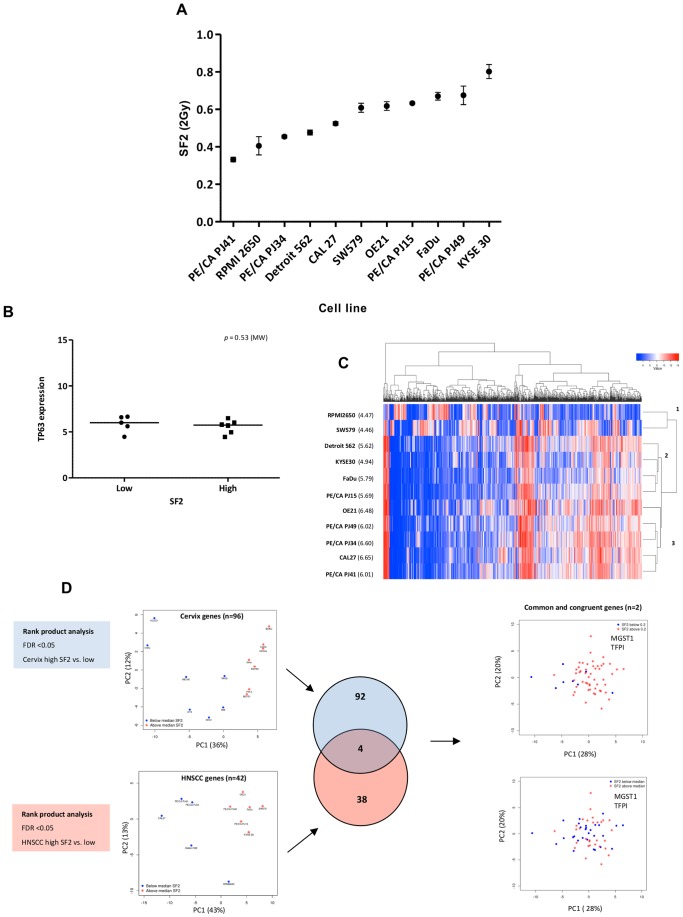
Characterisation of a head and neck squamous cell carcinoma (HNSCC) cell line cohort. **A)** Graph showing the mean SF2 (log10) (y-axis) for each of the 11 cervix cancer cell lines (x-axis). Error bars show the standard error of mean of 2–3 independent experiments. **B)** Graph showing that there is no difference in TP63 expression between the SF2 high and low groups. Bar shows the median expression. **C)** Unsupervised hierarchical clustering of the top 1000 genes ranked by coefficient of variation (from U133 array data). Heatmap colouring is by log_2_ expression value. Rows represent genes and columns are cell lines. x-axis dendrogram (clusters) indicates the similarity of the cell lines and y-axis dendrogram the similarity of genes. Cluster 1 represents two samples with the lowest TP63 values (p63 negative). Cluster 2 shows the grouping of the other p63− cell line, along with low TP63 expressing lines. Cluster 3 groups together all HNSCC lines with >6.0 (log2 expression) TP63 expression. **D)** Diagram to represent the integrated SF2 analysis of the cervix and HNSCC cell lines. Rank product analysis (FDR <0.05) identified 96 genes in the cervix cohort differentially expressed between SF2 low and high cell lines. An identical analysis in the HNSCC cell lines identifies 97 probesets (42 genes) differentially expressed between SF2 low and high cell lines. PCA of the cervix genes shows that they are capable of separating the cell lines by SF2. PCA of the HNSCC genes is equally capable of separating the samples based on SF2. The Venn diagram shows that only 4/138 genes are common between the two cohorts and of these only 2/138 are “congruent” and associated with the same directionality (high SF2/low SF2 in both HNSCC and cervix). PCA shows probeset expression of these two “common” and “congruent” genes (MGST1 and TFPI) in the NCI-60 dataset. The NCI-60 upper PCA shows data-points coloured for median SF2 and lower PCA coloured for 0.2, used previously to partition radiosensitive and radioresistant cell lines in this cohort.

Table 2 summarises the 11 HNSCC cell lines, which were all HPV negative (Figure S2). Although reported to be squamous cell carcinoma, three lacked p63 protein expression by Western blot (Figure S3), and had low transcript levels detected by microarray. The SF2 range (0.3–0.8) was similar to that for the cervix lines (Figure 4A), but the HNSCC cell lines were more radioresistant compared with the cervix (*p* = 0.003). The median SF2, used to partition the cell lines was 0.36 for cervix and 0.61 for HNSCC cell lines. As with cervix cell lines, there was no difference in SF2 between cell lines expressing high versus low levels of *TP63* (Figure 4B) and unsupervised hierarchical clustering partitioned the HNSCC cell lines into three groups reflecting *TP63* expression (Figure 4C). The most outlying cluster had the lowest *TP63* expression while the remaining two clusters divided the cell lines with expression </>6.0 (log_2_) TP63. These data show that both cervical carcinoma and HNSCC cell lines have similar radiosensitivities and global transcriptional profiles, with the majority of differences relating to the transcription factor p63. As such, the HNSCC cohort is a tissue-type distinct from cervix, but should be a good comparator for SF2 associated genes derived in cervical cell lines and *vice versa*.

**Table 2:** Summary characteristics of the head and neck cancer cell lines.

Cell Line*	Tumour sub-site*	Tumour Histotype*	SF2	HPV$	p63† (Western)	TP63† (array)
PE/CA PJ41	Oral squamous epithelium	SCC	0.33±0.01	–	+	6.01
RPMI2650	Nasal septum	SCC	0.41±0.03	–	–	4.47
PE/CA PJ34	Oral cavity	SCC	0.45±0.00	–	+	6.6
Detroit 562	Pharynx	SCC	0.48±0.01	–	+	5.62
CAL27	Tongue	SCC	0.52±0.01	–	+	6.65
SW579	Thyroid	SCC	0.61±0.01	–	–	4.46
OE21	Oesophagus	SCC	0.62±0.01	–	+	6.48
PE/CA PJ15	Tongue epithelium	SCC	0.63±0.01	–	+	5.69
FaDu	Pharynx	SCC	0.67±0.01	–	+	5.79
PE/CA PJ49	Tongue	SCC	0.68±0.02	–	+	6.02
KYSE 30	Oesophagus	SCC	0.80±0.02	–	–	4.97

* Provenance information was derived from ATCC including tissue origin and tumour histological type.

$ Table also includes the results of HPV genotyping (by HPV qRT-PCR),

† p63 expression from Western analysis and U133 plus 2.0 array derived TP63 expression values.

### Genes Differentially Expressed between High and Low SF2 Groups are Primarily Cell Type Specific and Cannot Stratify the NCI-60 Cell Lines

Differences between the cell lines partitioned using median SF2 were explored using genome-wide expression profiling. No differentially expressed transcripts were found by *LIMMA* following multiple-testing correction. This was also the case for linear models incorporating HPV and p63 expression as covariates, or in a 3-way ANOVA. While genes were identified that were differentially expressed (raw p<0.05), none passed false-discovery correction. An alternative method, Rank Product Analysis, applied to the cervix cell lines identified 96 differentially-expressed genes (pfp<0.01) (Table S3). These genes separated the cervix samples on the first principal component, accounting for 36% of the variation (Figure 4D), but could not separate the HNSCC cell lines based on SF2 (Figure S4A). A reciprocal analysis on the HNSCC lines identified a similar number of probesets (n = 97, mapping to 42 unique gene symbols, pfp<0.01) differentially expressed between high and low SF2 (Table S4). These genes performed well in separating the HNSCC cell lines (Figure 4D), but failed to separate the cervix lines (Figure S4B). This shows that the majority of the genes identified are cohort/tumour type specific. Only four (2.9%) of the 138 differentially expressed gene symbols were in both gene lists: *MGST1, IFITM2, TFPI* and *TGFB2*. Of these only two were congruent in being associated with radiosensitivity or radioresistance in both cohorts (*MGST1, TFPI*). Expression of these two genes did not separate the NCI-60 cell lines based on SF2 (Figure 4D). Similar results were achieved taking the convergence (n = 134) of the cervix and head and neck gene lists.

### Identification and Independent Validation of a Signature Associated with p63 Protein Expression

To test our signature generation approach, we applied the same methods (i.e. Rank product, mapping Exon 1.0ST gene-level data to U133 plus 2.0 array) to a more obvious biological phenotype: p63 protein expression. Rank product analysis identified genes differentially expressed between p63 positive and negative cell lines in both cervix (n = 395) and HNSCC (n = 335) cell lines pfp<0.01 (Figure S5A&B). Of these genes 62 were differentially expressed in both cell types and associated with p63 expression (Figure S5C). These common genes represent genes previously associated with squamous histology (e.g. KRT5, DSC3, CTA-55I10.1) [21]. Reassuringly, when this gene signature was applied to an independent dataset it could discriminate between adenocarcinoma and squamous cell carcinoma of the lung (non-small cell lung cancer) (Figure S6) [34]. There was little overlap between the p63 negative component of this signature and the adenocarcinoma signature applied previously (Figure 2). Given HeLa is the only adenocarcinoma cell line, this suggest that losing p63 expression is not the same transcriptionally as being ‘adenocarcinoma’. In terms of classification, it is predominantly the p63 positive component of the signature that facilitates separation in both cell lines and tumours. That said, as our methods could derive a signature capable of independent validation, SF2 appears to be a difficult phenotype to describe at the transcriptional level.

### Combined Analysis of Cervix and Head and Neck Cell Lines Increases Statistical Power, but Fails to Give Rise to a Robust Gene Signature Associated with SF2

Given our suspicion that there are only small transcriptional differences associated with the SF2 phenotype, we calculated the sample size required to detect transcriptional difference reflecting SF2 from the cervix cell line data. Using gene CTC-359D24.3 which had the largest standardised difference between group means of 2.48 (log_2_) combined with the smallest within group standard deviation (1.54) provides an optimistic estimate of required sample size for microarray classifiers [35]. This suggests that 27 samples (13 SF2 low and 14 SF2 high) would be required given the current spread of the data. The cohorts were combined to improve statistical power (n = 25). The two cohorts were split independently based on median SF2, as splitting on SF2 alone would create a bias between the cervix and HNSCC samples (Figure S7A). Samples below the median, whether cervix or head and neck were defined as radiosensitive and above the median were classified as radioresistant (Figure S7B). Twenty-two genes were differentially expressed between the SF2 high and low cohorts. These genes competently separated the cervix and HNSCC cell lines, with only one misclassification (Figure S7A), but did not separate the NCI-60 samples, whether separated on the median or 0.2 (Figure S8) [10]. This suggests that the data are potentially over-fitted and cannot generalise to the NCI-60 dataset. Interestingly 3/22 genes (KRT5, CSTA, FGFBP1) were identified as being associated with p63 previously and suggest an imbalance between histologies within the two SF2 groups. Repeating the analyses using the overall median for the combined cervix and HNSCC cell line cohort or the lowest quartile as a cut-off did not improve the discriminatory power in the three cell line cohorts. Similarly, pooling the cervix, HNSCC and NCI-60 cohorts did not work.

### Published Radiosensitivity Gene Signatures have a Varying Ability to Classify Cell Lines based on SF2

**Figure 5:**
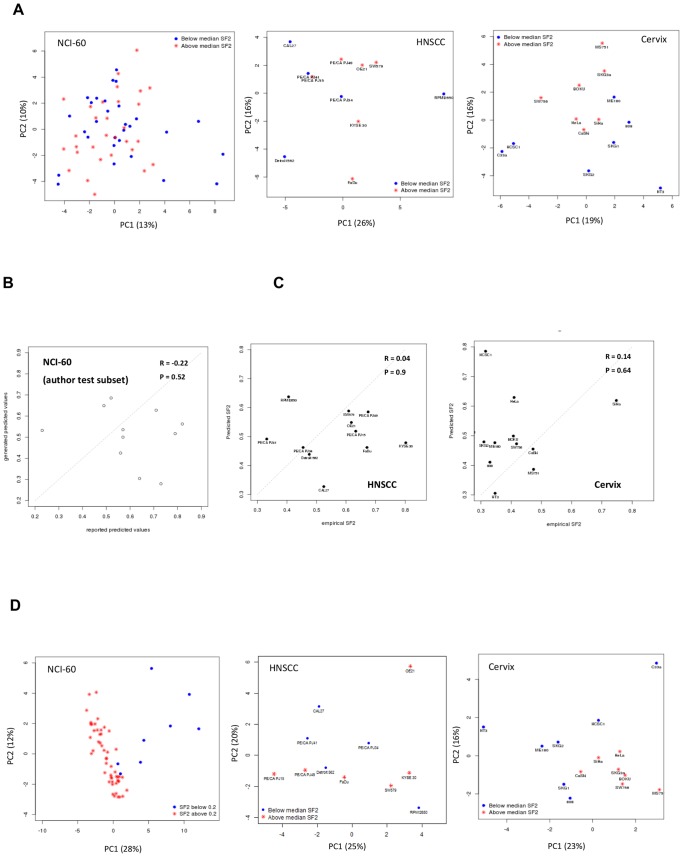
Assessment of established radiosensitivity gene signatures. **A)** PCA of the Tewari radiosensitivity gene signature. The original signature consists of 49 genes, with mapping to the NCI-60 (60 Plus2 probesets) HNSCC (60 Plus2 probesets) and cervix cell line (48/49 genes) datasets. The x-axis shows PC1, accounting for the largest amount of variation in the experiment and the y-axis shows the second principal component (PC2). Colouring based on median SF2, blue data-points are radiosensitive cell lines (below the median SF2) with red data-points being the radioresistant lines (above the median SF2). **B)** Implementation of the Eschrich radiosensitivity model [12]. Applied to a training set of 16 samples from the NCI-60 [13]. xy-scatterplot with the x-axis showing reported SF2 values, generated with these cell lines on a earlier array type (U95) against values generated by implementing the model in the current U133 plus 2.0 dataset (y-axis). Line indicates perfect correlation. **C)** Applied to the HNSCC and cervix cancer cell line cohorts. The y-axis indicates the predicted SF2 determined from the radiosensitivity model. The x-axis shows the empirically derived SF2 values. **D)** Principal component analysis of the Amundson radiosensitivity gene signature [10]. The original signature consists of 22 genes (33 Plus2 probesets), with mapping to the NCI-60 (33 Plus2 probesets), HNSCC (33 Plus2 probesets) and cervix cell line (21/22 genes) datasets. The x-axis shows PC1, accounting for the largest amount of variation in the experiment and the y-axis shows the second principal component (PC2). In the NCI-60 data colouring is based a threshold of 0.2 (previously defined [21] where the HNSCC and cervix cell line datasets are coloured by median SF2. In all cases blue data-points are radiosensitive cell lines (below the median SF2) with red data-points being the radioresistant lines (above the median SF2).

We also investigated published radiosensitivity gene signatures. Given that principal component analysis (PCA) gives an unsupervised/unbiased view of the major variation between different samples we used this method to assess how well a gene signature could separate samples based on SF2. First, we considered the Tewari signature derived by assessing cell viability in *in vitro* irradiated cervix tumour samples [8]. A 54 transcript signature mapped to 49 unique gene symbols partly separated the cervix (Figure 5A) but did not separate the HNSCC or NCI-60 cell lines into SF2 groupings (Figure 5A).

The Torres-Roca signature was trained on a historical microarray platform and when applied to the authors NCI-60 test subset [12] on a current array version (U133 plus 2.0) with standard normalisation (RMA), did not predict SF2 in the NCI-60 test subset (Figure 5B), the cervix lines or the HNSCC cells (Figure 5C). There was no statistically significant difference between the calculated radiosensitivity index (RSI) and the SF2 groupings in either the cervix (p = 0.74) or HNSCC (p = 0.32) cell lines (Figure S9) and no grouping by PCA when considering the gene expression values for the ten “hub” genes (Figure S10). In a subset of four published and described genes [12] there was a weak, but significant correlation between expression of *RPIA* (R = −0.3, *p*<0.01), *RBBP4* (R = −0.36, *p*<0.01), *RGS19* (R = −0.4, *p*<0.01) and SF2, in the NCI-60 (training) data. These genes however showed no association with SF2 in the cervix or HNSCC cohorts (Figure S11). Taken together our findings suggest that this gene signature, while capable of prognostication in clinical datasets [11] and successfully classifying cell lines based on SF2 in 5/12 (41.7%) cases, was not sufficiently robust to predict SF2 on the same cell lines on a different microarray platform or in independent cell line cohorts.

The last signature (Amundson) assessed was also derived using the NCI-60 panel and the basal expression of genes associated with SF2 [10]. Twenty-one genes partitioned the samples based on an SF2 threshold of 0.2. This 21-gene signature separated the NCI-60 cell lines according to SF2 along the first principal component (Figure 5D). These genes also partly separated the HNSCC cell lines using the second principal component. Similarly the cervix cell lines showed grouping based on SF2, using the first two principal components. These groupings are not ideal and could be optimised further, but this demonstrates for the first time that a gene signature trained on basal SF2 can be successfully applied to two independent cell line cohorts. This suggests that some genes associated with SF2 may generalise across cell-types, rather than being cohort/cell type specific.

## Discussion

Cell line-derived gene signatures have been successfully translated to clinical biomarkers that are both prognostic and predictive [36] and are particularly relevant in situations where measurements in primary tumours are difficult, as with radiosensitivity. Radiotherapy plays an important part in the management of cervix cancer and HNSCC and measurements of radiosensitivity have been shown to correlate with clinical radioresponse [3], [4]. The use of these tissue types is, however, currently under-represented when training signatures on radiosensitivity [10], [12].

As expected [30] the cervix cell line SF2 values varied but were independent of proliferation and plating efficiency. We also showed no association between SF2 and the expression of key cancer associated proteins. However, mTOR was >20-fold higher in radioresistant compared with radiosensitive cells and was moderately correlated with SF2 (R = 0.48, *p* = 0.08). High expression of mTOR protein was associated with a poor prognosis in cervical cancer treated with radiotherapy [37]. Therefore, mTOR may have a role in intrinsic radiosensitivity and clinical radioresponse and should be investigated further.

A rationale of this study was that radiosensitivity signatures might be improved if derived from homogeneous rather than heterogeneous cell line cohorts involving multiple tissues of origin and culture conditions. Despite the greater homogeneity of our cohort, there were key differences between the cell lines. Transcriptome analysis showed that C33a, commonly used as a model for HPV negative cervix cancer [38], is likely to be derived from small cell carcinoma and therefore may not be a good model, given that most cervix cancers are squamous cell in origin [39]. The epithelial marker p63 that can be lost in culture by squamous cells [18] was the most significant source of transcriptional variation between cervix cell lines. We also show that p63 has no association with SF2 in these cell lines. This result was also seen in HNSCC lines, showing that SF2 in two independent cohorts of cell lines is not associated with epithelial character.

HNSCC cell lines were similar to cervix cancer cell lines in their SF2 range, basal gene expression and partitioning based on p63 status. However, radiosensitivity signatures did not transfer between the tumour types. Only two genes (MGST1 and TFPI) were differentially expressed between low and high SF2 groups in both tumour types. There are a number of potential reasons for this finding. First, cell lines from different origins may have different mechanisms and consequential gene expression to deal with radiation-induced damage. This is supported by different tissues and their derivative cell lines having varying radiocurability and radiosensitivity [17]. However, cervix and head and neck cancer have broadly similar radiosensitivities and radiocurabilities. Second, the simple dichotomisation strategy applied (i.e. median partitioning of the cell lines) might not be the best approach, however previous work in clinical samples showed that median SF2 informed clinical radioresponse [3], [4], and repeating the analyses using a lower cut-off did not work. However, with a larger cohort, perhaps including the extremes of SF2, a different partitioning strategy might be more successful. Third, technical variation in measuring SF2 might be a problem, particularly with borderline samples. Fourth, differences in radiosensitivity (SF2) occur at a post-transcriptional level and protein-profiling methods may be more fruitful in deriving a radiosensitivity signature, although these rely on the availability and selection of appropriate antibodies [40]. Although there is interest in the protein expression of DNA damage response, the literature is conflicting with high expression associated with both good [41], [42] and poor [40] outcomes following radiotherapy. Another technical issue that might account for the lack of transferability of the signatures is the use of different platforms to measure gene expression. For example the methods for filtering applied to the Exon 1.0ST arrays used to generate the cervix signature cannot be applied to the U133 plus 2.0 arrays. However we show that this technical issue can be overcome in the generation of a p63 signature, using the same platforms (Figures S5 & S6).

Validation in an independent cohort is required to avoid over-training of gene signatures but has been rarely applied for radiosensitivity signatures. We tested three published signatures trained on either SF2 or viability 48 h following 3 Gy irradiation. The most developed signature trained on SF2 in the NCI-60 panel did not validate. However, the normalisation (MAS 5.0) and array type (HU6800) used in the original derivation and testing of the signature were different and this may account for the lack of reproducibility. This signature had been shown to be prognostic for radioresponse (locoregional control) and predictive of benefit from adjuvant radiotherapy in breast cancer patients [11]. Nevertheless, using the raw expression values alone or PCA transformation showed no separation of the three datasets; showing the signature is not sufficiently robust to transfer to other cell line datasets.

A signature derived in cervix tumours based on viability 48 h following 3 Gy irradiation [8] partly stratified the cervix lines, but did not separate the HNSCC or NCI-60 lines. This observation is consistent with our original hypothesis that radiosensitivity signatures might be more robust if trained on more homogeneous (and radiobiologically relevant) cell line cohorts. However, the NCI-60 trained Amundson signature separated the cervix and head and neck lines into high and low SF2 groups, albeit imperfectly. This finding does not support our original hypothesis but does suggest that further development of radiosensitivity signatures is worthwhile. The research area will benefit from expanding the number of cell line cohorts which have been well characterised and for which gene expression data are available. It is hoped that making our data publically available will aid further developments of radiosensitivity signatures.

In summary, our attempt to identify common transcripts associated with low and high SF2 measurements was not fruitful in a homogeneous single tumour type cell line cohort. We applied a relatively naïve approach to identify the genes associated with SF2. While it is likely that more advanced modelling of the data will result in a better understanding of the data and potentially reveal interesting candidate transcripts, this is beyond the scope of this paper. What is clear from these analyses is that intrinsic radiosensitivity, as measured by SF2, is a relatively subtle phenotype. The datasets generated in this study should benefit future work aimed at deriving a robust radiosensitivity signature. Our work suggests that a common transcriptional signature *can* reflect the radiosensitivity of tumours of heterogeneous origins, although much larger cohorts are required to overcome background noise.

## Supporting Information

Figure S1 **Unsupervised hierarchical clustering of protein-profiling data.** Pearson clustering of ZeptoMark data.
(DOCX)

Figure S2 **HNSCC lines are negative for expression of HPV E6/E7 or E2.** qRT-PCR for expression of viral oncogenes E6/E7 and E2 for HPV16 and HPV18.
(DOCX)

Figure S3 **Western blot showing p63 protein expression in the 11 HNSCC lines.** Blot shows p63 expression and Actin loading control.
(DOCX)

Figure S4 **Principal component analysis of the differentially expressed radiosensitivity genes (cervix and HNSCC lines).** Principal component analysis showing the separation of samples based on A). 96 genes differentially expressed between radiosensitive and radioresistant cervix lines. B). 42 genes differentially expressed between radiosensitive and radioresistant HNSCC.
(DOCX)

Figure S5 **Principal component analysis of differentially expressed genes between p63 positive and neagative cervix and HNSCC lines.** Supervised clustering; dendograms showing clustering of cervix and HNSCC cell lines based on p63 differentially expressed genes.
(DOCX)

Figure S6 **Principal component analysis showing independent validation of the p63 associated gene signature.** PCA showing the separation of lung cancer samples (NSCLC) into AC and SCC based on the expression of 62 genes commonly differentially expressed between cervix and HNSCC p63 positive and negative cell lines.
(DOCX)

Figure S7 **Combined cervix and HNSCC SF2 analysis.** Data showing the distribution of SF2 across the combined cohort and partitioning into two groups: SF2 high and SF2 low.
(DOCX)

Figure S8 **Genes from the combined analysis do not separate the NCI-60 dataset.** Data showing that the 22 genes identified by combining the cervix and HNSCC cohorts and repartitioning does not robustly partition the NCI60 cell lines.
(DOCX)

Figure S9 **Demonstration that the Eschrich model cannot be applied to the cervix or HNSCC samples.** Boxplots showing that the Eschrich model does not separate the cervix and HNSCC into statistically significantly different groups.
(DOCX)

Figure S10 **Principal component analysis of Eschrich model gene expression in multiple datasets.** Data shows that the Eschrich model gene expression does not successfully partition the NCI60, cervix or HNSCC datasets.
(DOCX)

Figure S11 **Behaviour of the top four Eschrich model genes in three datasets.** Data shows the gene expression of RP1A, RBBP4, RGS19 and ZNF208 in three datasets. Expression values (x-axis) are plotted against SF2 (y-axis). Line of best fit and R and p-values are displayed.
(DOCX)

Table S1 **Culture conditions for the cancer cell lines.** List of requirements for culture of the cervix and head and neck cell lines.
(DOCX)

Table S2 **Differentially expressed proteins from protein profiling array.** Results from LIMMA differential expression analysis of ZeptoMark protein-profiling arrays, comparing radiosensitive and radioresistant lines.
(DOCX)

Table S3 **Differentially expressed genes from Affymetrix Expression profiling (Exon 1.0ST arrays).** Results from Ranked Product differential expression analysis of cervix carcinoma cell lines profiling using Affymetrix expression 1.0ST arrays, comparing radiosensitive and radioresistant groups.
(DOCX)

Table S4 **Differentially expressed genes from Affymetrix Expression profiling (U133 plus 2.0).** Results from Ranked Product differential expression analysis of HNSCC cell lines profiling using Affymetrix U133 plus 2.0 expression arrays, comparing radiosensitive and radioresistant groups.
(DOCX)

## References

[1] Williams MV, Drinkwater KJ (2009) Radiotherapy in England in 2007: modelled demand and audited activity. Clin Oncol (R Coll Radiol) 21: 575–590. 19651499 10.1016/j.clon.2009.07.003

[2] Bristow RG, Hill RP (1990) Comparison between in vitro radiosensitivity and in vivo radioresponse in murine tumor cell lines. II: In vivo radioresponse following fractionated treatment and in vitro/in vivo correlations. Int J Radiat Oncol Biol Phys 18: 331–345. 2303364 10.1016/0360-3016(90)90098-5

[3] West CM, Davidson SE, Roberts SA, Hunter RD (1997) The independence of intrinsic radiosensitivity as a prognostic factor for patient response to radiotherapy of carcinoma of the cervix. Br J Cancer 76: 1184–1190. 9365167 10.1038/bjc.1997.531 PMC2228123

[4] Bjork-Eriksson T, West C, Karlsson E, Mercke C (2000) Tumor radiosensitivity (SF2) is a prognostic factor for local control in head and neck cancers. Int J Radiat Oncol Biol Phys 46: 13–19. 10656366 10.1016/s0360-3016(99)00373-9

[5] West CM, Hendry JH (1992) Intrinsic radiosensitivity as a predictor of patient response to radiotherapy. BJR Suppl 24: 146–152. 1290690

[6] Otomo T, Hishii M, Arai H, Sato K, Sasai K (2004) Microarray analysis of temporal gene responses to ionizing radiation in two glioblastoma cell lines: up-regulation of DNA repair genes. J Radiat Res 45: 53–60. 15133290 10.1269/jrr.45.53

[7] Chung YM, Kim BG, Park CS, Huh SJ, Kim J, et al (2005) Increased expression of ICAM-3 is associated with radiation resistance in cervical cancer. Int J Cancer 117: 194–201. 15880373 10.1002/ijc.21180

[8] Tewari D, Monk BJ, Al-Ghazi MS, Parker R, Heck JD, et al (2005) Gene expression profiling of in vitro radiation resistance in cervical carcinoma: a feasibility study. Gynecol Oncol 99: 84–91. 16109440 10.1016/j.ygyno.2005.05.043

[9] Lorenzi PL, Reinhold WC, Varma S, Hutchinson AA, Pommier Y, et al (2009) DNA fingerprinting of the NCI-60 cell line panel. Mol Cancer Ther 8: 713–724. 19372543 10.1158/1535-7163.MCT-08-0921 PMC4020356

[10] Amundson SA, Do KT, Vinikoor LC, Lee RA, Koch-Paiz CA, et al (2008) Integrating global gene expression and radiation survival parameters across the 60 cell lines of the National Cancer Institute Anticancer Drug Screen. Cancer Res 68: 415–424. 18199535 10.1158/0008-5472.CAN-07-2120

[11] Eschrich S, Fulp WJ, Pawitan Y, Foekens JA, Smid M, et al (2012) Validation of a Radiosensitivity Molecular Signature in Breast Cancer. Clin Cancer Res 18: 5134–5143. 22832933 10.1158/1078-0432.CCR-12-0891 PMC3993974

[12] Eschrich S, Zhang H, Zhao H, Boulware D, Lee JH, et al (2009) Systems biology modeling of the radiation sensitivity network: a biomarker discovery platform. Int J Radiat Oncol Biol Phys 75: 497–505. 19735874 10.1016/j.ijrobp.2009.05.056 PMC2762403

[13] Eschrich SA, Pramana J, Zhang H, Zhao H, Boulware D, et al (2009) A gene expression model of intrinsic tumor radiosensitivity: prediction of response and prognosis after chemoradiation. Int J Radiat Oncol Biol Phys 75: 489–496. 19735873 10.1016/j.ijrobp.2009.06.014 PMC3038688

[14] Torres-Roca JF, Eschrich S, Zhao H, Bloom G, Sung J, et al (2005) Prediction of radiation sensitivity using a gene expression classifier. Cancer Res 65: 7169–7176. 16103067 10.1158/0008-5472.CAN-05-0656

[15] Eschrich SA, Fulp WJ, Pawitan Y, Foekens JA, Smid M, et al (2012) Validation of a radiosensitivity molecular signature in breast cancer. Clin Cancer Res 18: 5134–5143. 22832933 10.1158/1078-0432.CCR-12-0891 PMC3993974

[16] Kim HS, Kim SC, Kim SJ, Park CH, Jeung HC, et al (2012) Identification of a radiosensitivity signature using integrative metaanalysis of published microarray data for NCI-60 cancer cells. BMC genomics 13: 348. 22846430 10.1186/1471-2164-13-348 PMC3472294

[17] Fertil B, Malaise EP (1985) Intrinsic radiosensitivity of human cell lines is correlated with radioresponsiveness of human tumors: analysis of 101 published survival curves. Int J Radiat Oncol Biol Phys 11: 1699–1707. 4030437 10.1016/0360-3016(85)90223-8

[18] Barbieri CE, Tang LJ, Brown KA, Pietenpol JA (2006) Loss of p63 leads to increased cell migration and up-regulation of genes involved in invasion and metastasis. Cancer Res 66: 7589–7597. 16885358 10.1158/0008-5472.CAN-06-2020

[19] Brady CS, Bartholomew JS, Burt DJ, Duggan-Keen MF, Glenville S, et al (2000) Multiple mechanisms underlie HLA dysregulation in cervical cancer. Tissue Antigens 55: 401–411. 10885560 10.1034/j.1399-0039.2000.550502.x

[20] Marples B, Longhurst D, Eastham AM, West CM (1998) The ratio of initial/residual DNA damage predicts intrinsic radiosensitivity in seven cervix carcinoma cell lines. Br J Cancer 77: 1108–1114. 9569047 10.1038/bjc.1998.184 PMC2150146

[21] Hall JS, Leong HS, Armenoult LS, Newton GE, Valentine HR, et al (2011) Exon-array profiling unlocks clinically and biologically relevant gene signatures from formalin-fixed paraffin-embedded tumour samples. Br J Cancer 104: 971–981. 21407225 10.1038/bjc.2011.66 PMC3065290

[22] Hall JS, Iype R, Armenoult LS, Taylor J, Miller CJ, et al (2013) Poor Prognosis Associated With Human Papillomavirus alpha7 Genotypes in Cervical Carcinoma Cannot Be Explained by Intrinsic Radiosensitivity. Int J Radiat Oncol Biol Phys 85: e223–229. 23332225 10.1016/j.ijrobp.2012.11.030

[23] Pawlak M, Schick E, Bopp MA, Schneider MJ, Oroszlan P, et al (2002) Zeptosens’ protein microarrays: a novel high performance microarray platform for low abundance protein analysis. Proteomics 2: 383–393. 12164697 10.1002/1615-9861(200204)2:4<383::AID-PROT383>3.0.CO;2-E

[24] Begg AC, Stewart FA, Vens C (2011) Strategies to improve radiotherapy with targeted drugs. Nat Rev Cancer 11: 239–253. 21430696 10.1038/nrc3007

[25] Irizarry RA, Hobbs B, Collin F, Beazer-Barclay YD, Antonellis KJ, et al (2003) Exploration, normalization, and summaries of high density oligonucleotide array probe level data. Biostatistics 4: 249–264. 12925520 10.1093/biostatistics/4.2.249

[26] Yates T, Okoniewski MJ, Miller CJ (2008) X:Map: annotation and visualization of genome structure for Affymetrix exon array analysis. Nucleic Acids Res 36: D780–786. 17932061 10.1093/nar/gkm779 PMC2238884

[27] Smyth GK (2004) Linear models and empirical bayes methods for assessing differential expression in microarray experiments. Stat Appl Genet Mol Biol 3: Article3. 16646809 10.2202/1544-6115.1027

[28] Roberts SA (1990) DRFIT: a program for fitting radiation survival models. Int J Radiat Biol 57: 1243–1246. 1971847 10.1080/09553009014551321

[29] Benjamini Y, Hochberg Y (1995) Controlling the false discovery rate: a practical and powerful approach to multiple testing. Journal of the Royal Statistical Society Series B 57: 289–300.

[30] Banath JP, Macphail SH, Olive PL (2004) Radiation sensitivity, H2AX phosphorylation, and kinetics of repair of DNA strand breaks in irradiated cervical cancer cell lines. Cancer Res 64: 7144–7149. 15466212 10.1158/0008-5472.CAN-04-1433

[31] Pedersen N, Mortensen S, Sorensen SB, Pedersen MW, Rieneck K, et al (2003) Transcriptional gene expression profiling of small cell lung cancer cells. Cancer Res 63: 1943–1953. 12702587

[32] Senoo M, Pinto F, Crum CP, McKeon F (2007) p63 Is essential for the proliferative potential of stem cells in stratified epithelia. Cell 129: 523–536. 17482546 10.1016/j.cell.2007.02.045

[33] Crook T, Wrede D, Vousden KH (1991) p53 point mutation in HPV negative human cervical carcinoma cell lines. Oncogene 6: 873–875. 1646990

[34] Dobbin E, Corrigan PM, Walsh CP, Welham MJ, Freeburn RW, et al (2008) Tel/PDGFRbeta inhibits self-renewal and directs myelomonocytic differentiation of ES cells. Leuk Res 32: 1554–1564. 18355917 10.1016/j.leukres.2008.02.007

[35] Dobbin KK, Zhao Y, Simon RM (2008) How large a training set is needed to develop a classifier for microarray data? Clin Cancer Res 14: 108–114. 18172259 10.1158/1078-0432.CCR-07-0443

[36] Toustrup K, Sorensen BS, Alsner J, Overgaard J (2012) Hypoxia gene expression signatures as prognostic and predictive markers in head and neck radiotherapy. Semin Radiat Oncol 22: 119–127. 22385919 10.1016/j.semradonc.2011.12.006

[37] Kim MK, Kim TJ, Sung CO, Choi CH, Lee JW, et al (2010) High expression of mTOR is associated with radiation resistance in cervical cancer. J Gynecol Oncol 21: 181–185. 20922141 10.3802/jgo.2010.21.3.181 PMC2948226

[38] Hampson L, El Hady ES, Moore JV, Kitchener H, Hampson IN (2001) The HPV16 E6 and E7 proteins and the radiation resistance of cervical carcinoma. FASEB J 15: 1445–1447. 11387252 10.1096/fj.00-0728fje

[39] de Sanjose S, Quint WG, Alemany L, Geraets DT, Klaustermeier JE, et al (2010) Human papillomavirus genotype attribution in invasive cervical cancer: a retrospective cross-sectional worldwide study. Lancet Oncol 11: 1048–1056. 20952254 10.1016/S1470-2045(10)70230-8

[40] Moeller BJ, Yordy JS, Williams MD, Giri U, Raju U, et al (2011) DNA repair biomarker profiling of head and neck cancer: Ku80 expression predicts locoregional failure and death following radiotherapy. Clin Cancer Res 17: 2035–2043. 21349997 10.1158/1078-0432.CCR-10-2641 PMC3092475

[41] Choudhury A, Nelson LD, Teo MT, Chilka S, Bhattarai S, et al (2010) MRE11 expression is predictive of cause-specific survival following radical radiotherapy for muscle-invasive bladder cancer. Cancer Res 70: 7017–7026. 20843819 10.1158/0008-5472.CAN-10-1202 PMC2941719

[42] Pavon MA, Parreno M, Leon X, Sancho FJ, Cespedes MV, et al (2008) Ku70 predicts response and primary tumor recurrence after therapy in locally advanced head and neck cancer. Int J Cancer 123: 1068–1079. 18546291 10.1002/ijc.23635

