# Supplementary figures and images for: Investigation of Radiosensitivity Gene Signatures in Cancer Cell Lines
Source: PLoS One. 2014 Jan 22;9(1):e86329. doi: 10.1371/journal.pone.0086329 (PMC3899227; doi:10.1371/journal.pone.0086329)

**Figure S1:** Unsupervised clustering of the ZeptoMARK protein profiling data.


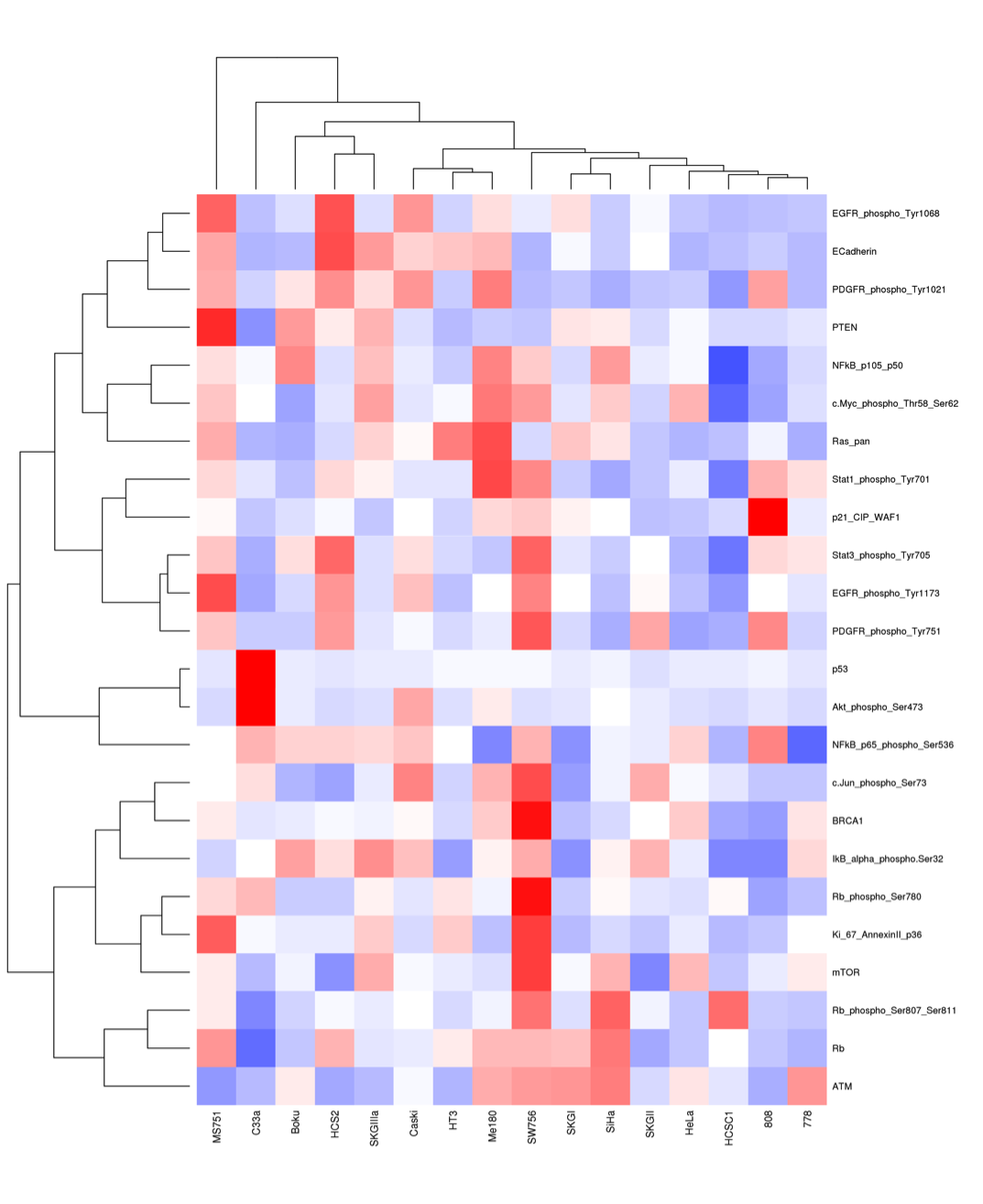

Supplement: Figure S1 — Unsupervised hierarchical clustering of protein-profiling data. Pearson clustering of ZeptoMark data. (DOCX) [file pone.0086329.s001.docx]

**Figure S9:** Implementation of the Eschrich model, RSI partitioned by empirical SF2 grouping.


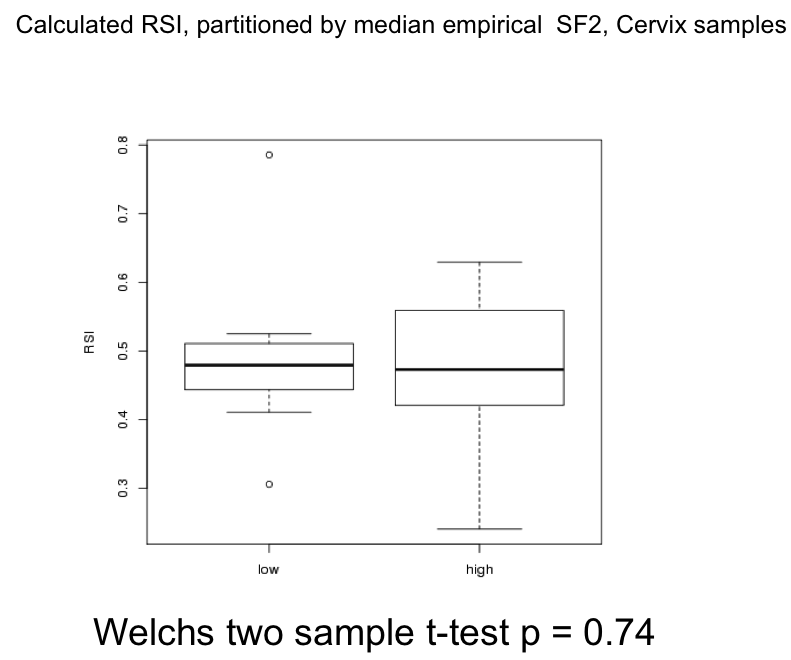


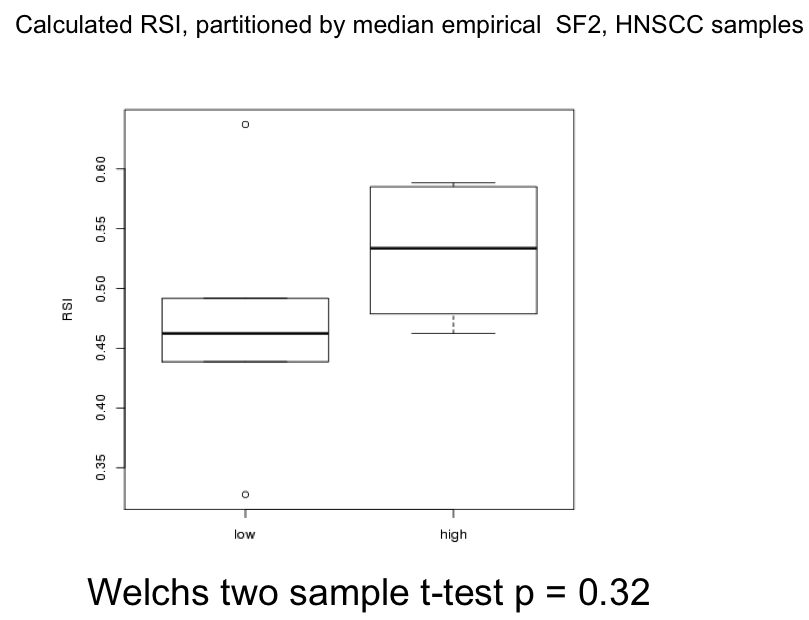

Supplement: Figure S9 — Demonstration that the Eschrich model cannot be applied to the cervix or HNSCC samples. Boxplots showing that the Eschrich model does not separate the cervix and HNSCC into statistically significantly different groups. (DOCX) [file pone.0086329.s009.docx]

**Figure S11:** Behaviour of the top four Eschrich model genes in three datasets.


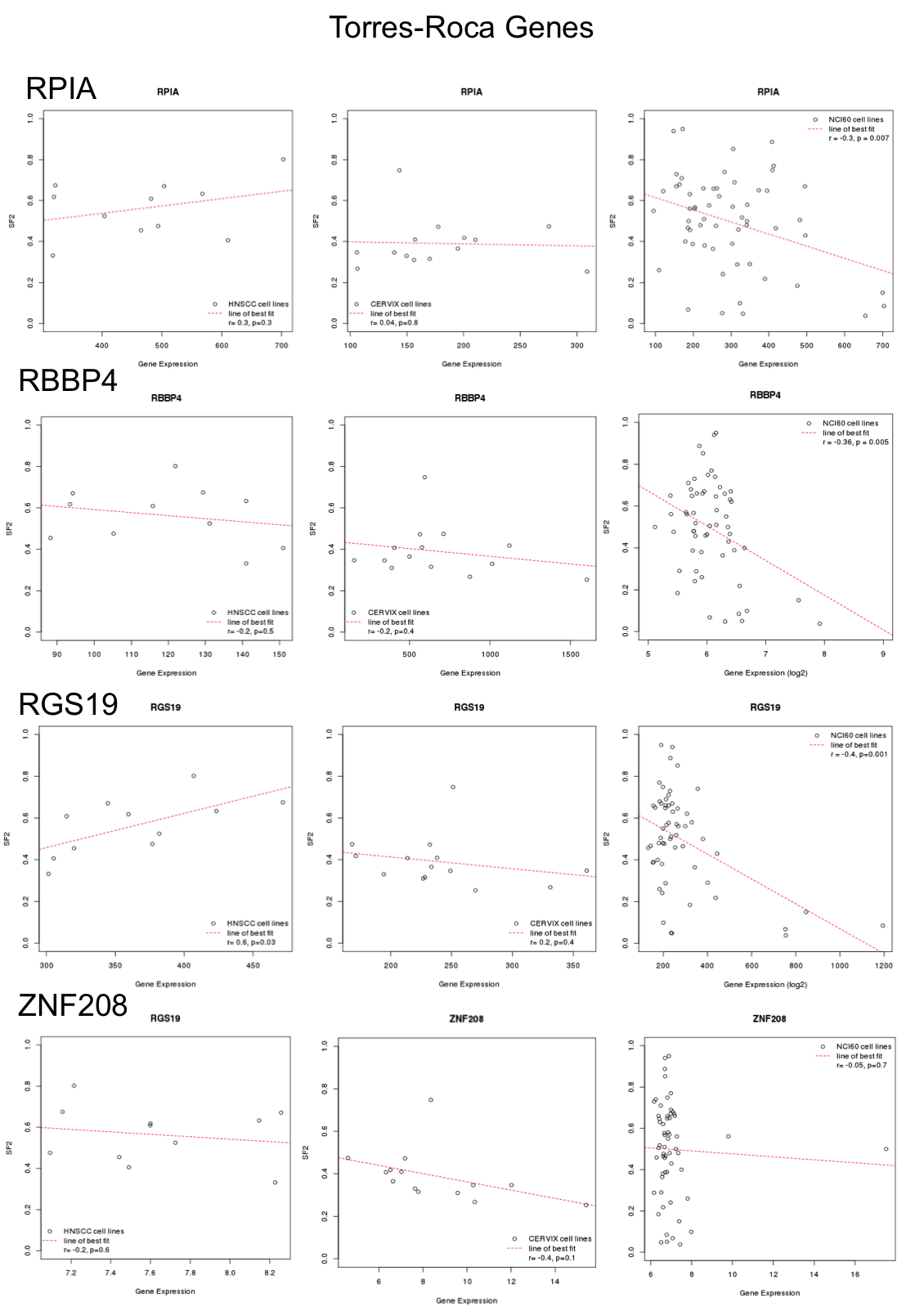

Supplement: Figure S11 — Behaviour of the top four Eschrich model genes in three datasets. Data shows the gene expression of RP1A, RBBP4, RGS19 and ZNF208 in three datasets. Expression values (x-axis) are plotted against SF2 (y-axis). Line of best fit and R and p-values are displayed. (DOCX) [file pone.0086329.s011.docx]
