# Supplementary material for: Investigation of Radiosensitivity Gene Signatures in Cancer Cell Lines
Source: PLoS One. 2014 Jan 22;9(1):e86329. doi: 10.1371/journal.pone.0086329 (PMC3899227; doi:10.1371/journal.pone.0086329)

**Figure S2.** HNSCC cell lines are HPV negative.

HPV RNA genotyping. qRT-PCR to determine expression of viral oncogenes E2, E6 and E7, for HPV16 and HPV18. Controls: C33a is a HPV negative cell line, SiHa is a HPV16 positive cell line and 778 is a HPV18 positive cell line. –RT is a negative batch control for the HNSCC cell lines. Any expression >40Ct was considered positive. Graph shows the average of three technical replicates, where controls were accurately determined and HNSCC lines were negative for HPV16 and HPV18 oncogene expression.


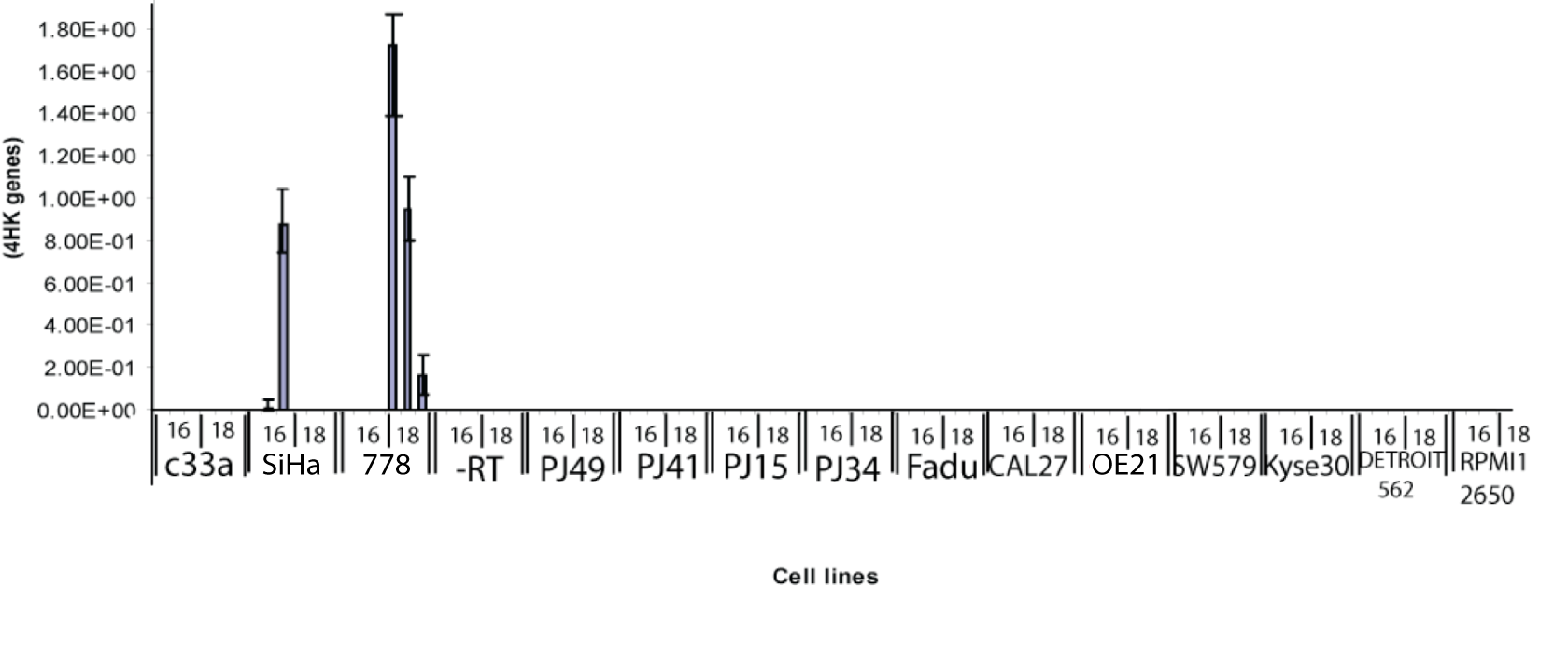

Supplement: Figure S2 — HNSCC lines are negative for expression of HPV E6/E7 or E2. qRT-PCR for expression of viral oncogenes E6/E7 and E2 for HPV16 and HPV18. (DOCX) [file pone.0086329.s002.docx]
