# Supplementary material for: Investigation of Radiosensitivity Gene Signatures in Cancer Cell Lines
Source: PLoS One. 2014 Jan 22;9(1):e86329. doi: 10.1371/journal.pone.0086329 (PMC3899227; doi:10.1371/journal.pone.0086329)
Supplement: Figure S3 — Western blot showing p63 protein expression in the 11 HNSCC lines. Blot shows p63 expression and Actin loading control. (DOCX) [file pone.0086329.s003.docx]

**Figure S3.** Western blot showing p63 expression in the 11 HNSCC cell lines. Actin was used as a loading control.
