# Supplementary material for: Investigation of Radiosensitivity Gene Signatures in Cancer Cell Lines
Source: PLoS One. 2014 Jan 22;9(1):e86329. doi: 10.1371/journal.pone.0086329 (PMC3899227; doi:10.1371/journal.pone.0086329)

**Figure S4 A):** Principal component analysis of the H&N cell lines separated by 96 cervix genes.


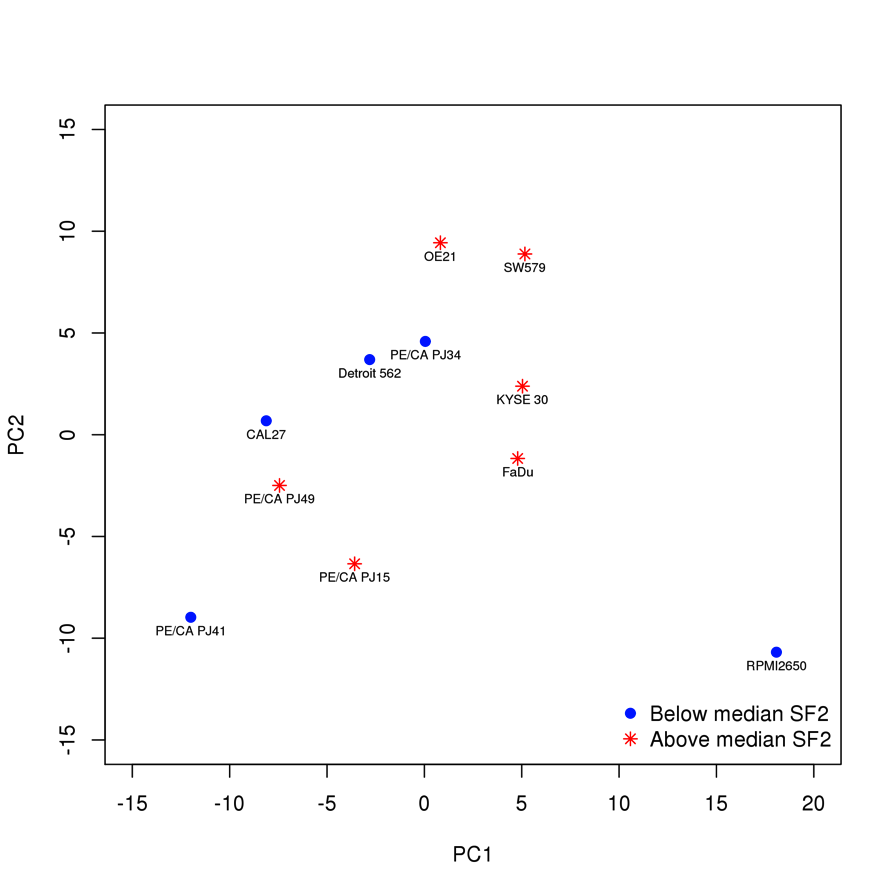


**Figure S4 B):** Principal component analysis of the cervix cell lines separated by 42 HNSCC SF2 genes


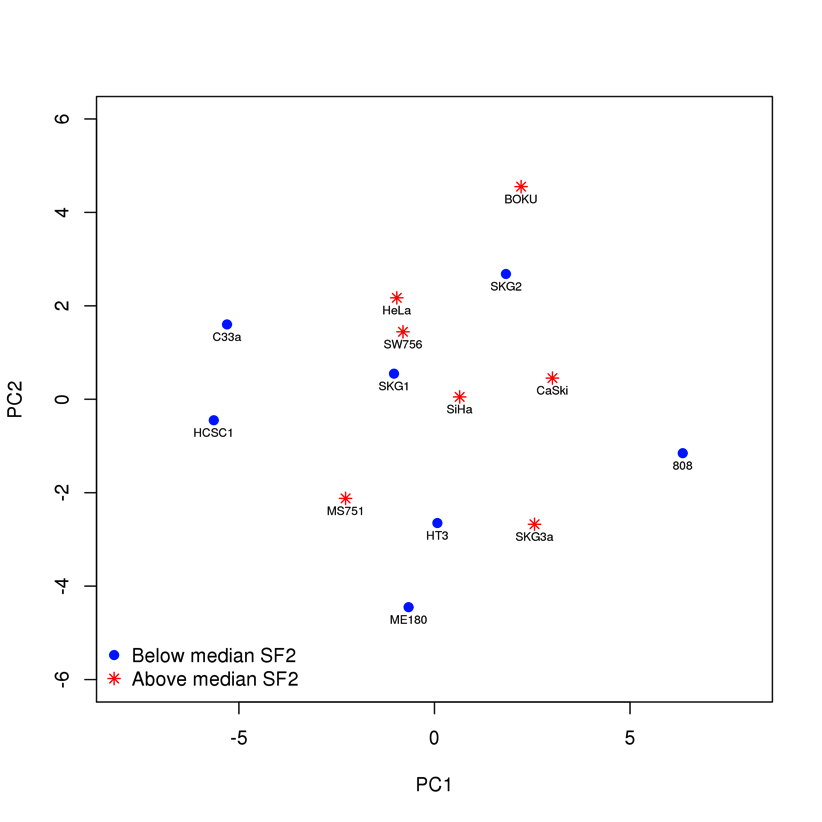

Supplement: Figure S4 — Principal component analysis of the differentially expressed radiosensitivity genes (cervix and HNSCC lines). Principal component analysis showing the separation of samples based on A). 96 genes differentially expressed between radiosensitive and radioresistant cervix lines. B). 42 genes differentially expressed between radiosensitive and radioresistant HNSCC. (DOCX) [file pone.0086329.s004.docx]
