# Supplementary material for: Investigation of Radiosensitivity Gene Signatures in Cancer Cell Lines
Source: PLoS One. 2014 Jan 22;9(1):e86329. doi: 10.1371/journal.pone.0086329 (PMC3899227; doi:10.1371/journal.pone.0086329)
Supplement: Figure S5 — Principal component analysis of differentially expressed genes between p63 positive and neagative cervix and HNSCC lines. Supervised clustering; dendograms showing clustering of cervix and HNSCC cell lines based on p63 differentially expressed genes. (DOCX) [file pone.0086329.s005.docx]

**Figure S5:** p63 as a positive control phenotype for gene signature generation. A). Identifications of genes differentially expressed (Rank product) between p63 positive and negative cervix cell lines (pfp<0.01) B). Identifications of genes differentially expressed between p63 positive and negative head and neck cell lines (pfp<0.01) C). Table of 62 common and congruent differentially expressed (DE) Genes determined by Rank Product Analysis

P63 negative (western)

P63 positive (western)

**A)
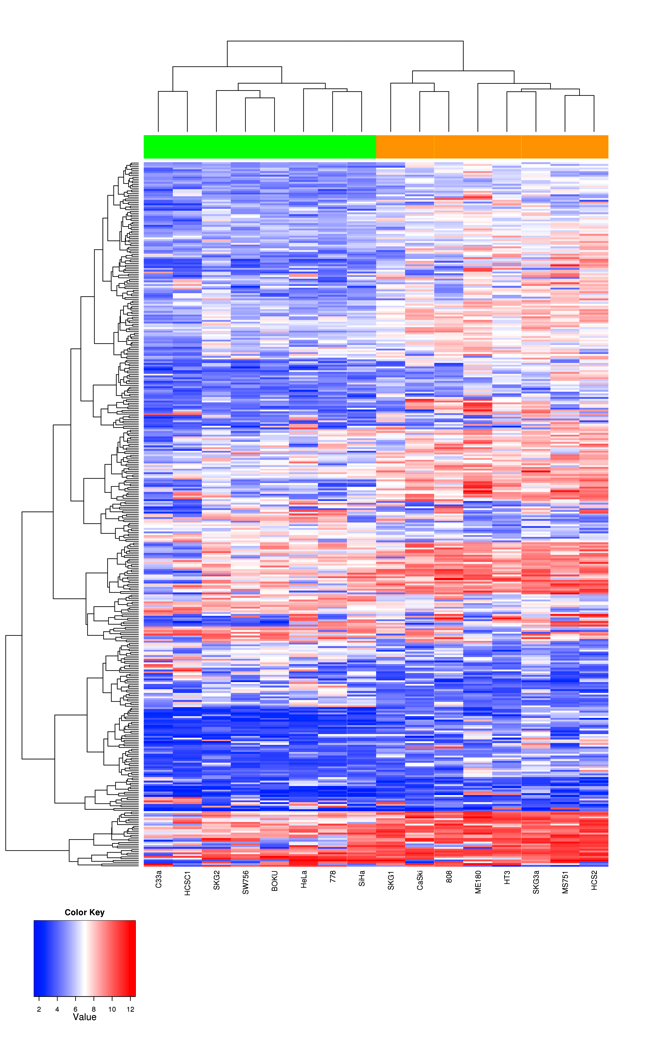
B)
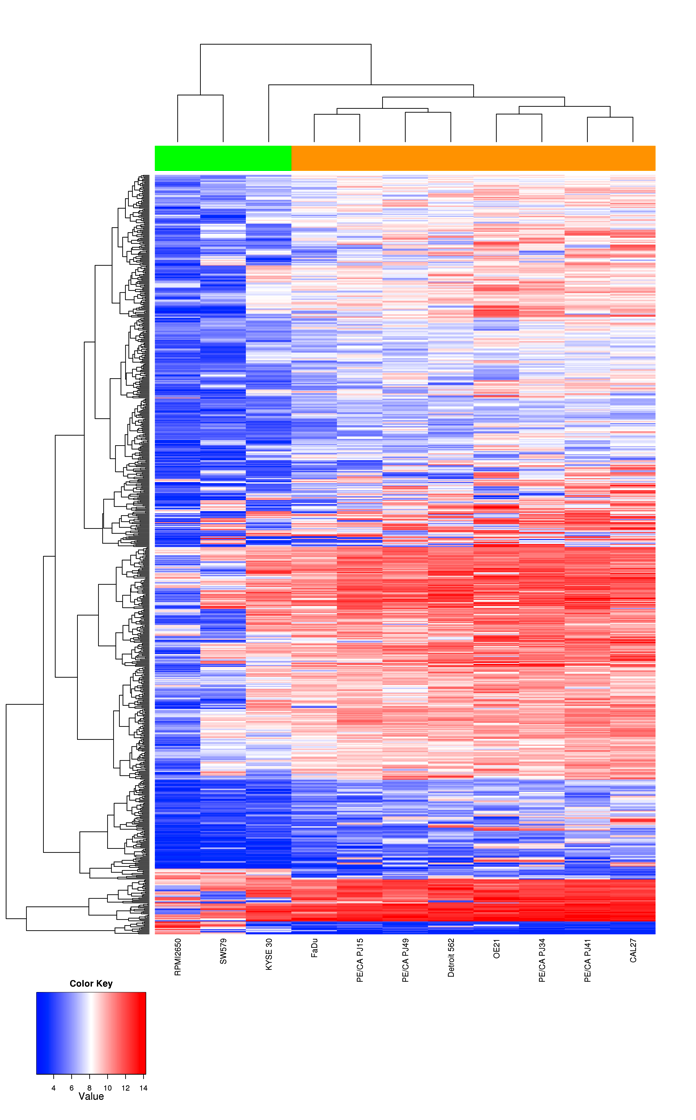
**

**C)**

| CTA-55I10.1  CSTA  TP63  SERPINB13  DSC3  DSG3  ESRP1  KRT5  DSC2  CDH1  CLCA2  KRT13  IL1A  ITGB6  OLR1 | SFN  DSP  KYNU  IRF6  GPX2  GRHL2  TMEM30B  LPAR3  MAL2  PRRG4  TRIM29  ZNF750  PTHLH  F11R  CD44  GALNT3 | RASEF  LAMC2  GJB5  S100A14  MAST4  ITGA6  IL8  THBD  CXCL16  LPAR6  SAMD9  TACSTD2  IFI16  IGFBP3  IFI44  GM2A | FGFBP1  DAPP1  FYB  IL20RB  IRX2  CASP1  IL1RAP  SOX15  CA2  KCNJ15  SERPINB4  C10orf116  PDPN  ZNF165  BNC1 |
| --- | --- | --- | --- |
