# Supplementary material for: Investigation of Radiosensitivity Gene Signatures in Cancer Cell Lines
Source: PLoS One. 2014 Jan 22;9(1):e86329. doi: 10.1371/journal.pone.0086329 (PMC3899227; doi:10.1371/journal.pone.0086329)
Supplement: Figure S6 — Principal component analysis showing independent validation of the p63 associated gene signature. PCA showing the separation of lung cancer samples (NSCLC) into AC and SCC based on the expression of 62 genes commonly differentially expressed between cervix and HNSCC p63 positive and negative cell lines. (DOCX) [file pone.0086329.s006.docx]

**Figure S6:** Ability of the p63 gene signature (62 genes) to separate non-small cell lung cancer (NSCLC) samples into AC (red) and SCC (blue) groups. This independent dataset (Kuner et al, 2009) is on U133 plus 2.0 arrays (analogous to the NCI-60 data) and contains four described misclassifications. We have shown previously that a gene signature derived from cervix FFPE can separate these samples (Hall et al, BJC, 2011), this demonstrates this with a signature derived from cervix and head and neck cell lines.


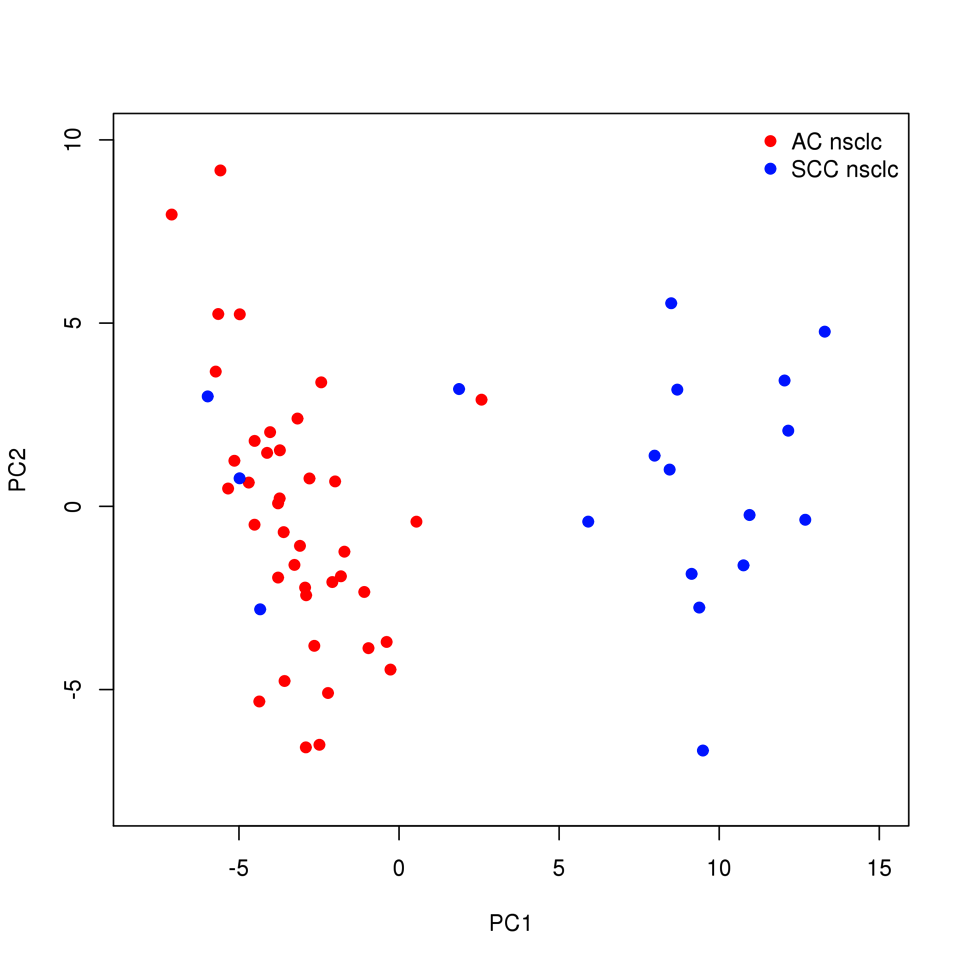


(31% variance)

(10.3% variance)

Kuner R, Muley T, Meister M, Ruschhaupt M, Buness A, Xu EC, Schnabel P, Warth A, Poustka A, Sultmann H, Hoffmann H (2009) Global gene expression analysis reveals specific patterns of cell junctions in non-small cell lung cancer subtypes. Lung Cancer 63: 32–38
