# Supplementary material for: Investigation of Radiosensitivity Gene Signatures in Cancer Cell Lines
Source: PLoS One. 2014 Jan 22;9(1):e86329. doi: 10.1371/journal.pone.0086329 (PMC3899227; doi:10.1371/journal.pone.0086329)

**Figure S7:** Combined cervix and head and neck cell line SF2 analysis. **A)** Demonstrates that a bias would exist that splits the samples into predominately SF2 low (cervix) and SF2 high (HNSCC). This is most likely to identify genes associated with cell of origin, which are indistinguishable with SF2. **B)** Cervix and HNSCC cohorts were split on the median and divided into SF2 low and SF2 high.

**A**


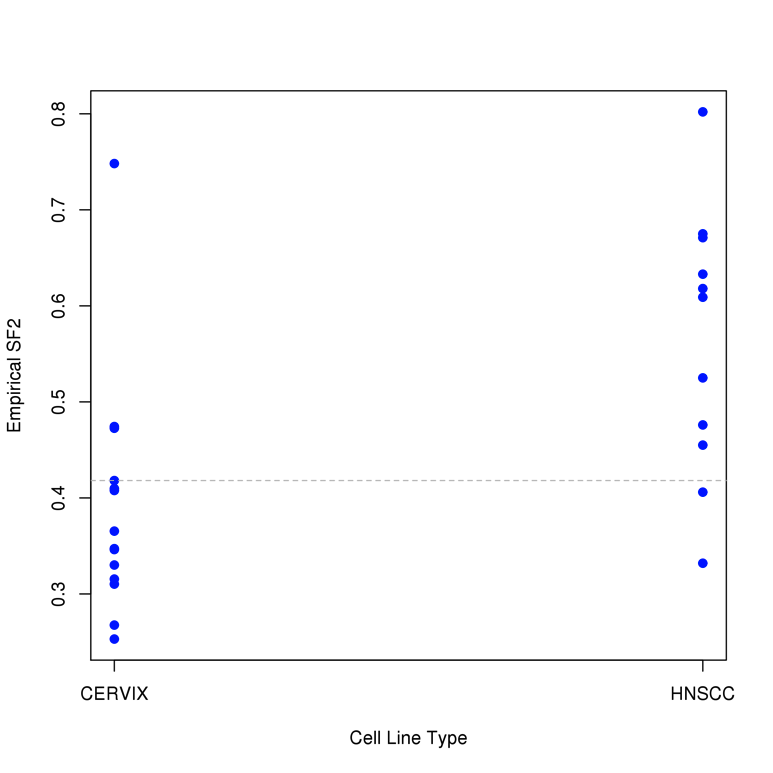


**B**


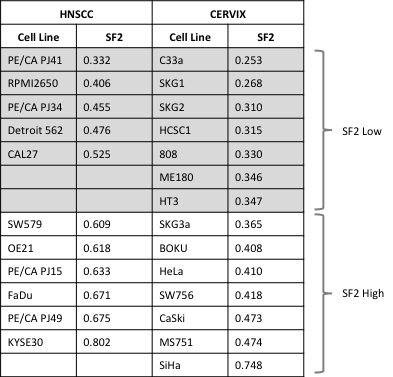

Supplement: Figure S7 — Combined cervix and HNSCC SF2 analysis. Data showing the distribution of SF2 across the combined cohort and partitioning into two groups: SF2 high and SF2 low. (DOCX) [file pone.0086329.s007.docx]
