# Supplementary material for: Investigation of Radiosensitivity Gene Signatures in Cancer Cell Lines
Source: PLoS One. 2014 Jan 22;9(1):e86329. doi: 10.1371/journal.pone.0086329 (PMC3899227; doi:10.1371/journal.pone.0086329)
Supplement: Figure S8 — Genes from the combined analysis do not separate the NCI-60 dataset. Data showing that the 22 genes identified by combining the cervix and HNSCC cohorts and repartitioning does not robustly partition the NCI60 cell lines. (DOCX) [file pone.0086329.s008.docx]

**Figure S8:** Combined cervix and head and neck cell line SF2 analysis. **A)** PCA showing good separation on the first principle component of the cell lines based on SF2 (variance = 39.8%). 22 genes pass pfp <0.01. Only a single misclassification exists.

**A)**

| Upregulated in Low SF2 | Upregulated in High SF2 |
| --- | --- |
| MGST1 | EHF |
| GNG11 | KRT5 |
| SPINT2 | TATDN1 |
| TM4SF1 | SPARC |
| NNMT | GJB6 |
| TFPI2 | PTGS2 |
| TGFBI | RP11-165J3.6 |
| FAM26F | CSTA |
| ZNF83 | FGFBP1 |
| CAV1 | CADM1 |
| LY6K |  |
| ALDH1A3 |  |


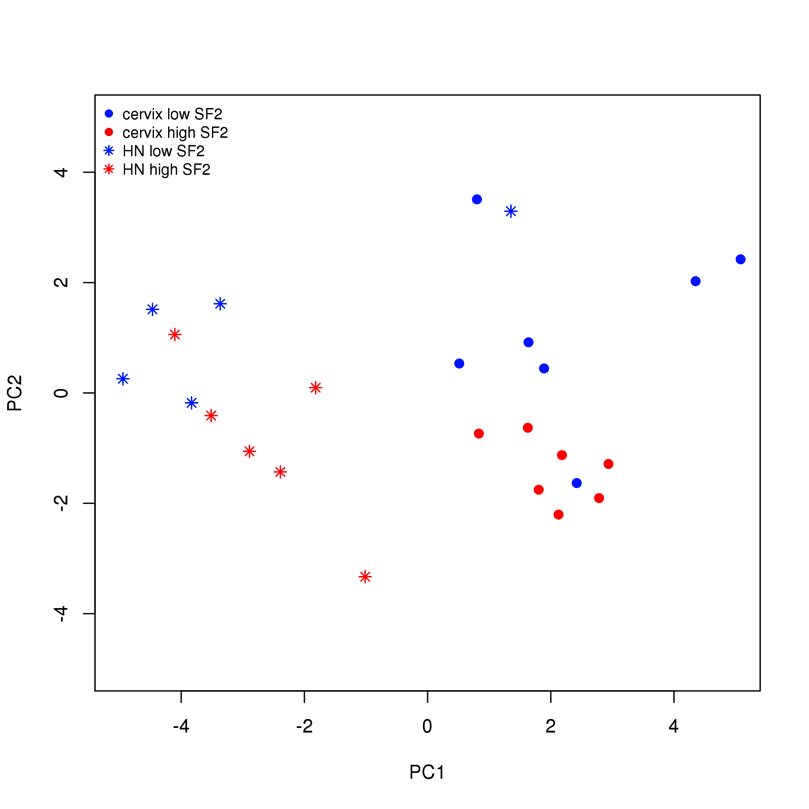


(39.8%)

(13.9%)

**B)**
