# Supplementary material for: Investigation of Radiosensitivity Gene Signatures in Cancer Cell Lines
Source: PLoS One. 2014 Jan 22;9(1):e86329. doi: 10.1371/journal.pone.0086329 (PMC3899227; doi:10.1371/journal.pone.0086329)
Supplement: Figure S10 — Principal component analysis of Eschrich model gene expression in multiple datasets. Data shows that the Eschrich model gene expression does not successfully partition the NCI60, cervix or HNSCC datasets. (DOCX) [file pone.0086329.s010.docx]

**Figure S10:** Principal component analysis of the Eschrich model genes (n=21/22 genes) in multiple datasets.


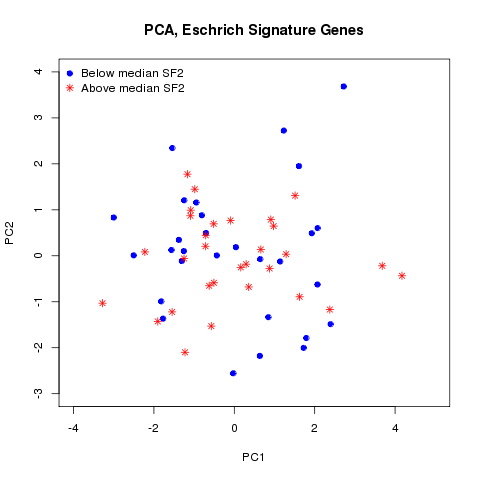

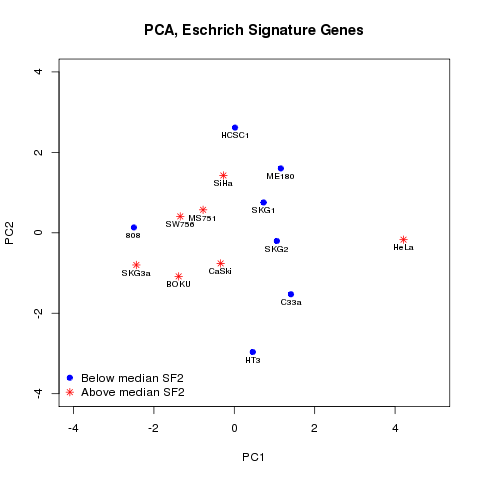

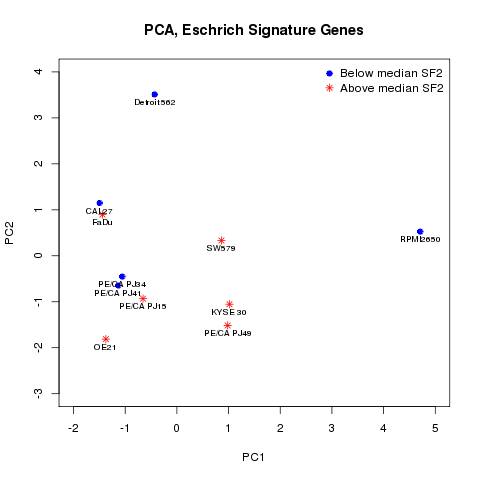


HNSCC

NCI60

CERVIX
