# Supplementary material for: Investigation of Radiosensitivity Gene Signatures in Cancer Cell Lines
Source: PLoS One. 2014 Jan 22;9(1):e86329. doi: 10.1371/journal.pone.0086329 (PMC3899227; doi:10.1371/journal.pone.0086329)
Supplement: Table S1 — Culture conditions for the cancer cell lines. List of requirements for culture of the cervix and head and neck cell lines. (DOCX) [file pone.0086329.s012.docx]

**Table S1.** Table showing the culture requirements (media) for the cervix and head and neck cell lines.

| **Cell line** | **Media** |
| --- | --- |
| Cervix cell lines | DMEM + Glutamax + 10% FCS |
| **Cell line** | **Media** |
| PE/CA PJ41 | IMDM + 2mM Glutamine + 10% FCS |
| RPMI2650 | EMEM + 2mM Glutamine +10% FCS |
| PE/CA PJ34 | IMDM + 2mM Glutamine + 10% FCS |
| Detroit 562 | EMEM + 2mM Glutamine + 1% NEAA +10% FCS |
| CAL27 | DMEM/F12 (1:1) + 2mM Glutamine + 10% FCS |
| SW579 | RPMI 1640 +2mM Glutamine + 10% FCS |
| OE21 | RPMI 1640 + 2mM Glutamine + 10% FCS |
| PE/CA PJ15 | IMDM + 2mM Glutamine + 10% FCS |
| FaDu | EMEM + 2mM Glutamine + 1mM sodium pyruvate + 1% NEAA +10% FCS |
| PE/CA PJ49 | IMDM + 2mM Glutamine + 10% FCS |
| KYSE 30 | RPMI/Hams-F12(1:1) + 2mM glutamine + 10% FCS |
