# Supplementary material for: Investigation of Radiosensitivity Gene Signatures in Cancer Cell Lines
Source: PLoS One. 2014 Jan 22;9(1):e86329. doi: 10.1371/journal.pone.0086329 (PMC3899227; doi:10.1371/journal.pone.0086329)
Supplement: Table S2 — Differentially expressed proteins from protein profiling array. Results from LIMMA differential expression analysis of ZeptoMark protein-profiling arrays, comparing radiosensitive and radioresistant lines. (DOCX) [file pone.0086329.s013.docx]

**Table S2.** LIMMA differential expression analysis of protein profiling data (ZeptoMARK)

| **ID** | **Log2 FC** | **t** | **P.Value** | **FDR corrected p** |
| --- | --- | --- | --- | --- |
| mTOR | 0.042 | 3.530 | 0.004 | 0.091 |
| PTEN | 0.561 | 2.387 | 0.033 | 0.295 |
| NFkB_p105_p50 | 0.052 | 2.276 | 0.041 | 0.295 |
| IkB_alpha_phospho.Ser32 | 0.006 | 2.174 | 0.049 | 0.295 |
| Ki_67_AnnexinII_p36 | 0.098 | 2.033 | 0.063 | 0.304 |
| Stat3_phospho_Tyr705 | 0.059 | 1.698 | 0.114 | 0.390 |
| BRCA1 | 0.029 | 1.774 | 0.100 | 0.390 |
| c.Myc_phospho_Thr58_Ser62 | 0.083 | 1.546 | 0.147 | 0.426 |
| EGFR_phospho_Tyr1173 | 0.021 | 1.458 | 0.169 | 0.426 |
| NFkB_p65_phospho_Ser536 | 0.005 | 1.427 | 0.178 | 0.426 |
| EGFR_phospho_Tyr1068 | 0.019 | 0.971 | 0.350 | 0.645 |
| Rb_phospho_Ser780 | 0.019 | 0.917 | 0.376 | 0.645 |
| p53 | -0.007 | -1.058 | 0.310 | 0.645 |
| Rb_phospho_Ser807_Ser811 | 0.005 | 1.013 | 0.330 | 0.645 |
| Akt_phospho_Ser473 | -0.096 | -0.743 | 0.471 | 0.665 |
| c.Jun_phospho_Ser73 | 0.005 | 0.771 | 0.455 | 0.665 |
| Rb | 0.002 | 0.827 | 0.424 | 0.665 |
| ATM | 0.007 | 0.613 | 0.551 | 0.670 |
| p21_CIP_WAF1 | -0.003 | -0.621 | 0.545 | 0.670 |
| Ras_pan | -0.003 | -0.601 | 0.558 | 0.670 |
| PDGFR_phospho_Tyr1021 | 0.000 | 0.263 | 0.796 | 0.910 |
| ECadherin | 0.025 | 0.167 | 0.870 | 0.949 |
| Stat1_phospho_Tyr701 | 0.001 | 0.038 | 0.971 | 0.987 |
| PDGFR_phospho_Tyr751 | 0.000 | 0.017 | 0.987 | 0.987 |

Log_2_ fold change is: high SF2 – low SF2.
