# Supplementary material for: Investigation of Radiosensitivity Gene Signatures in Cancer Cell Lines
Source: PLoS One. 2014 Jan 22;9(1):e86329. doi: 10.1371/journal.pone.0086329 (PMC3899227; doi:10.1371/journal.pone.0086329)
Supplement: Table S3 — Differentially expressed genes from Affymetrix Expression profiling (Exon 1.0ST arrays). Results from Ranked Product differential expression analysis of cervix carcinoma cell lines profiling using Affymetrix expression 1.0ST arrays, comparing radiosensitive and radioresistant groups. (DOCX) [file pone.0086329.s014.docx]

**Table S3.** 96 genes differentially expressed between cervix SF2- low and high cell lines groups by Ranked Product Analysis (pfp <0.01)

| **Gene Symbol** | **Computed Rank Product** | **Log2 Fold change (low/high)** | **pfp** | **P.value** |
| --- | --- | --- | --- | --- |
| **Up-regulated in SF2 low** | | | | |
| FAM26F | 122.8195 | 0.1117 | 0 | <0.001 |
| RP1-93H18.6 | 183.3985 | 0.1386 | 0 | <0.001 |
| MGST1 | 206.1733 | 0.1298 | 0 | <0.001 |
| KRT7 | 261.0606 | 0.1417 | 0 | <0.001 |
| ARHGEF5L | 308.3792 | 0.1707 | 0 | <0.001 |
| TDRD9 | 421.3424 | 0.172 | 0 | <0.001 |
| AC018641.6 | 422.4798 | 0.1796 | 0 | <0.001 |
| KYNU | 487.9441 | 0.2325 | 4.00E-04 | <0.001 |
| NEFH | 492.6501 | 0.2652 | 3.00E-04 | <0.001 |
| SYCP2 | 494.0714 | 0.2884 | 3.00E-04 | <0.001 |
| CPS1 | 497.8949 | 0.2239 | 3.00E-04 | <0.001 |
| SPINK6 | 518.0855 | 0.3321 | 2.00E-04 | <0.001 |
| LY6K | 521.2751 | 0.1825 | 2.00E-04 | <0.001 |
| MET | 567.5844 | 0.213 | 4.00E-04 | <0.001 |
| SLC43A3 | 609.1973 | 0.2363 | 4.00E-04 | <0.001 |
| AC092535.2 | 644.2437 | 0.2857 | 6.00E-04 | <0.001 |
| CYR61 | 670.8972 | 0.2616 | 6.00E-04 | <0.001 |
| Z99716.1 | 677.7564 | 0.2833 | 7.00E-04 | <0.001 |
| IL6 | 698.301 | 0.2601 | 8.00E-04 | <0.001 |
| HIST1H3C | 710.7674 | 0.2702 | 8.00E-04 | <0.001 |
| TM4SF1 | 785.3033 | 0.2631 | 0.0019 | <0.001 |
| AL049651.1 | 785.4748 | 0.2643 | 0.0018 | <0.001 |
| AP001527.2 | 789.1416 | 0.2981 | 0.0018 | <0.001 |
| NRIP3 | 793.5524 | 0.333 | 0.0018 | <0.001 |
| CTB-161A2.3 | 794.0201 | 0.2886 | 0.0017 | <0.001 |
| AC016738.4 | 818.5894 | 0.2948 | 0.0023 | <0.001 |
| SNX7 | 846.313 | 0.2475 | 0.0027 | <0.001 |
| GNG11 | 852.1387 | 0.3427 | 0.0027 | <0.001 |
| TNFRSF11B | 857.6568 | 0.2943 | 0.0027 | <0.001 |
| RNU4ATAC | 857.6718 | 0.3331 | 0.0026 | <0.001 |
| IFITM2 | 867.3888 | 0.2199 | 0.0026 | <0.001 |
| TFPI | 879.5428 | 0.3103 | 0.0028 | <0.001 |
| C2orf27A | 880.2852 | 0.3115 | 0.0027 | <0.001 |
| CACNA2D1 | 892.968 | 0.3569 | 0.0029 | <0.001 |
| FSTL1 | 901.4956 | 0.2878 | 0.0029 | <0.001 |
| PRTFDC1 | 912.5095 | 0.33 | 0.0029 | <0.001 |
| AKR1C3 | 914.4482 | 0.3934 | 0.0029 | <0.001 |
| MPV17L | 918.132 | 0.3094 | 0.0029 | <0.001 |
| FAM7A3 | 919.7985 | 0.3075 | 0.0028 | <0.001 |
| TGFB2 | 921.4917 | 0.3006 | 0.0028 | <0.001 |
| ACSL5 | 928.8445 | 0.3026 | 0.0028 | <0.001 |
| SMC1B | 934.0585 | 0.358 | 0.0028 | <0.001 |
| PLK2 | 946.466 | 0.2535 | 0.0029 | <0.001 |
| MT1P2 | 947.3792 | 0.3157 | 0.0029 | <0.001 |
| RP11-124N14.3 | 956.569 | 0.5832 | 0.003 | <0.001 |
| PDE7B | 960.9771 | 0.338 | 0.003 | <0.001 |
| RP11-528L24.2 | 961.0125 | 0.2888 | 0.003 | <0.001 |
| GLIPR1 | 962.6217 | 0.3081 | 0.0029 | <0.001 |
| CDH13 | 966.8346 | 0.3481 | 0.0031 | <0.001 |
| BDNF | 978.9988 | 0.3276 | 0.0032 | <0.001 |
| CYP1B1 | 981.8086 | 0.2842 | 0.0032 | <0.001 |
| RP11-799O21.1 | 982.4896 | 0.3149 | 0.0032 | <0.001 |
| ZNF83 | 991.0393 | 0.3411 | 0.0033 | <0.001 |
| UCHL1 | 1013.7198 | 0.4974 | 0.0036 | <0.001 |
| RP11-893F2.4 | 1026.6411 | 0.321 | 0.0038 | <0.001 |
| NHLRC1 | 1037.4631 | 0.313 | 0.004 | <0.001 |
| PVRL3 | 1051.2449 | 0.3325 | 0.0043 | <0.001 |
| LDHC | 1062.839 | 0.349 | 0.0044 | <0.001 |
| PZP | 1067.1662 | 0.3107 | 0.0044 | <0.001 |
| CFHR1 | 1096.2184 | 0.3451 | 0.0049 | <0.001 |
| ARHGAP29 | 1100.767 | 0.2999 | 0.0049 | <0.001 |
| CLIP4 | 1110.8956 | 0.3099 | 0.005 | <0.001 |
| CPNE8 | 1114.7683 | 0.2843 | 0.0049 | <0.001 |
| EPB41L2 | 1116.0353 | 0.3373 | 0.0049 | <0.001 |
| AC003991.3 | 1118.273 | 0.4628 | 0.0049 | <0.001 |
| ZNF300 | 1118.4048 | 0.4127 | 0.0048 | <0.001 |
| NMU | 1119.1311 | 0.342 | 0.0048 | <0.001 |
| BST2 | 1130.8466 | 0.3453 | 0.005 | <0.001 |
| AP000872.1 | 1132.7197 | 0.3124 | 0.005 | <0.001 |
| CLEC2B | 1137.6162 | 0.4057 | 0.005 | <0.001 |
| XIST | 1138.9864 | 0.4781 | 0.005 | <0.001 |
| THEM4 | 1157.6378 | 0.3142 | 0.0056 | <0.001 |
| RP11-346D6.6 | 1163.5496 | 0.4499 | 0.0058 | <0.001 |
| AL109823.3 | 1174.0561 | 0.3516 | 0.0059 | <0.001 |
| USMG5P1 | 1183.62 | 0.3595 | 0.006 | <0.001 |
| NNMT | 1186.7621 | 0.3162 | 0.0061 | <0.001 |
| ADAMTS1 | 1195.8388 | 0.3554 | 0.0062 | <0.001 |
| CFH | 1235.6956 | 0.3897 | 0.0074 | <0.001 |
| NQO1 | 1243.637 | 0.2765 | 0.0077 | <0.001 |
| AL359711.1 | 1253.8426 | 0.3735 | 0.0079 | <0.001 |
| AF127577.10 | 1255.265 | 0.3427 | 0.0079 | <0.001 |
| SERPINB1 | 1256.5429 | 0.3203 | 0.0079 | <0.001 |
| **Up-regulated in SF2 high** | | | | |
| CTC-359D24.3 | 64.2054 | 14.2215 | <0.001 | <0.001 |
| SNORD64 | 267.5412 | 5.9683 | <0.001 | <0.001 |
| SNORD116-20 | 334.4644 | 4.9614 | <0.001 | <0.001 |
| CADM1 | 384.9928 | 5.853 | <0.001 | <0.001 |
| ALDH1A1 | 406.5609 | 5.8991 | <0.001 | <0.001 |
| GJA1 | 615.9878 | 2.6431 | 0.0015 | <0.001 |
| AC011484.3 | 692.5185 | 3.321 | 0.0027 | <0.001 |
| SLC46A3 | 771.387 | 3.0981 | 0.0044 | <0.001 |
| C8orf42 | 784.1089 | 2.7256 | 0.0043 | <0.001 |
| GPX2 | 801.5808 | 3.0952 | 0.0043 | <0.001 |
| GTSF1 | 820.3404 | 4.2127 | 0.0044 | <0.001 |
| FBN2 | 832.2985 | 2.9889 | 0.0042 | <0.001 |
| SNORD116-14 | 883.2069 | 3.0716 | 0.0056 | <0.001 |
| TOX | 946.8076 | 2.9825 | 0.0089 | <0.001 |

Median gene signal, per sample was used to calculate differential expression. Exon array mapping performed in Annmap. Log2 fold change (low/high) indicates the magnitude difference between classes. Pfp indicates percent false positive taking into account false discovery rate correction. P-value is a uncorrected p-value.
