# Supplementary material for: Investigation of Radiosensitivity Gene Signatures in Cancer Cell Lines
Source: PLoS One. 2014 Jan 22;9(1):e86329. doi: 10.1371/journal.pone.0086329 (PMC3899227; doi:10.1371/journal.pone.0086329)
Supplement: Table S4 — Differentially expressed genes from Affymetrix Expression profiling (U133 plus 2.0). Results from Ranked Product differential expression analysis of HNSCC cell lines profiling using Affymetrix U133 plus 2.0 expression arrays, comparing radiosensitive and radioresistant groups. (DOCX) [file pone.0086329.s015.docx]

**Table S4.** 97 probesets mapping to 42 Genes differentially expressed between HNSCC SF2- low and high cell lines groups by Ranked Product Analysis (FDR p<0.05)

| **Plus2 probeset ID** | **Gene Symbol^§^** | **Computed Rank Product** | **Log2 Fold change (low/high)** | **pfp** | **P.value** |
| --- | --- | --- | --- | --- | --- |
| **Up-regulated in SF2 Low** | | | | | |
| 213423_x_at | TUSC3 | 268.8042 | 0.0704 | 0 | <0.001 |
| 202363_at | SPOCK1 | 369.9189 | 0.1047 | 0 | <0.001 |
| 209228_x_at | TUSC3 | 405.3124 | 0.0969 | 0 | <0.001 |
| 242546_at | NULL | 422.7643 | 0.141 | 0 | <0.001 |
| 214240_at | GAL | 524.8417 | 0.1766 | 0.001 | <0.001 |
| 209278_s_at | TFPI2 | 548.7942 | 0.1707 | 8.00E-04 | <0.001 |
| 242881_x_at | NULL | 565.3151 | 0.1566 | 0.001 | <0.001 |
| 231882_at | FLJ39632 | 565.5802 | 0.1761 | 9.00E-04 | <0.001 |
| 204540_at | EEF1A2 | 608.7079 | 0.2066 | 0.0018 | <0.001 |
| 1565162_s_at | MGST1 | 632.7732 | 0.1836 | 0.0016 | <0.001 |
| 204733_at | KLK6 | 659.9667 | 0.1855 | 0.0018 | <0.001 |
| 212298_at | NRP1 | 664.3737 | 0.1463 | 0.0017 | <0.001 |
| 206067_s_at | WT1 | 745.1465 | 0.1745 | 0.0023 | <0.001 |
| 239010_at | NULL | 747.1671 | 0.2087 | 0.0022 | <0.001 |
| 231736_x_at | MGST1 | 751.7283 | 0.1952 | 0.0022 | <0.001 |
| 224918_x_at | MGST1 | 783.0643 | 0.1886 | 0.0022 | <0.001 |
| 214612_x_at | MAGEA6 | 786.142 | 0.2213 | 0.0022 | <0.001 |
| 201427_s_at | SEPP1 | 923.1611 | 0.1856 | 0.0041 | <0.001 |
| 209227_at | TUSC3 | 989.1585 | 0.2064 | 0.0054 | <0.001 |
| 226834_at | NULL | 1016.9079 | 0.2203 | 0.0058 | <0.001 |
| 1569582_at | LOC201651 | 1051.4643 | 0.2179 | 0.0067 | <0.001 |
| 202628_s_at | SERPINE1 | 1060.4587 | 0.2386 | 0.0065 | <0.001 |
| 209942_x_at | MAGEA3 | 1069.8529 | 0.2478 | 0.0067 | <0.001 |
| 213258_at | TFPI | 1141.1531 | 0.2906 | 0.0086 | <0.001 |
| 201426_s_at | VIM | 1148.8029 | 0.3063 | 0.0085 | <0.001 |
| 202627_s_at | SERPINE1 | 1176.8326 | 0.2362 | 0.0096 | <0.001 |
| 228116_at | NULL | 1181.8649 | 0.2967 | 0.0093 | <0.001 |
| **Up-regulated in SF2 High** | | | | | |
| 225645_at | EHF | 142.669 | 22.2418 | 0 | <0.001 |
| 219850_s_at | EHF | 143.3215 | 22.7496 | 0 | <0.001 |
| 224189_x_at | EHF | 190.2892 | 18.1833 | 0 | <0.001 |
| 222717_at | SDPR | 308.0653 | 10.8561 | 0 | <0.001 |
| 206504_at | CYP24A1 | 360.4184 | 10.7252 | 4.00E-04 | <0.001 |
| 202917_s_at | S100A8 | 381.288 | 9.939 | 5.00E-04 | <0.001 |
| 203535_at | S100A9 | 394.5295 | 10.0442 | 4.00E-04 | <0.001 |
| 222802_at | EDN1 | 429.8529 | 10.5876 | 4.00E-04 | <0.001 |
| 232361_s_at | EHF | 443.8673 | 9.1631 | 4.00E-04 | <0.001 |
| 200665_s_at | NULL | 458.56 | 6.5835 | 5.00E-04 | <0.001 |
| 212531_at | LCN2 | 523.9258 | 7.2399 | 0.0012 | <0.001 |
| 222932_at | EHF | 529.5795 | 7.9472 | 0.0012 | <0.001 |
| 210004_at | OLR1 | 531.361 | 7.362 | 0.0012 | <0.001 |
| 201650_at | KRT19 | 545.6165 | 7.1075 | 0.0013 | <0.001 |
| 218182_s_at | CLDN1 | 572.9219 | 7.7988 | 0.0014 | <0.001 |
| 203423_at | RBP1 | 585.6005 | 8.1469 | 0.0015 | <0.001 |
| 205476_at | CCL20 | 620.4917 | 7.2088 | 0.0021 | <0.001 |
| 209909_s_at | TGFB2 | 620.7407 | 8.0672 | 0.0019 | <0.001 |
| 223861_at | HORMAD1 | 636.9295 | 7.1587 | 0.0019 | <0.001 |
| 219630_at | PDZK1IP1 | 642.2834 | 7.7778 | 0.0019 | <0.001 |
| 201820_at | KRT5 | 677.2019 | 8.8617 | 0.0024 | <0.001 |
| 1553589_a_at | PDZK1IP1 | 691.6346 | 7.3975 | 0.0024 | <0.001 |
| 222549_at | CLDN1 | 699.0701 | 7.0968 | 0.0023 | <0.001 |
| 232360_at | EHF | 703.8524 | 6.3738 | 0.0023 | <0.001 |
| 204259_at | MMP7 | 753.1919 | 6.0379 | 0.0033 | <0.001 |
| 231771_at | GJB6 | 761.3285 | 4.3106 | 0.0032 | <0.001 |
| 202688_at | TNFSF10 | 808.9914 | 4.1581 | 0.0038 | <0.001 |
| 213293_s_at | TRIM22 | 814.8657 | 6.6174 | 0.0038 | <0.001 |
| 212768_s_at | OLFM4 | 840.9178 | 8.1811 | 0.0044 | <0.001 |
| 228121_at | TGFB2 | 842.1725 | 6.5345 | 0.0043 | <0.001 |
| 220407_s_at | TGFB2 | 844.4543 | 6.4153 | 0.0042 | <0.001 |
| 211906_s_at | SERPINB4 | 878.538 | 6.4604 | 0.0047 | <0.001 |
| 224009_x_at | DHRS9 | 880.5029 | 6.2942 | 0.0045 | <0.001 |
| 202357_s_at | CFB | 887.3386 | 5.5584 | 0.0046 | <0.001 |
| 244050_at | PTPLAD2 | 893.8494 | 5.6386 | 0.0048 | <0.001 |
| 212143_s_at | IGFBP3 | 898.2855 | 3.1182 | 0.0047 | <0.001 |
| 213849_s_at | PPP2R2B | 903.9443 | 5.9276 | 0.0047 | <0.001 |
| 225911_at | NPNT | 919.4857 | 5.8107 | 0.005 | <0.001 |
| 202086_at | MX1 | 922.0859 | 5.7296 | 0.0049 | <0.001 |
| 202687_s_at | TNFSF10 | 923.2236 | 4.1572 | 0.0048 | <0.001 |
| 226374_at | CXADR | 932.919 | 6.2026 | 0.0049 | <0.001 |
| 242873_at | NULL | 933.2068 | 4.9063 | 0.0048 | <0.001 |
| 212667_at | NULL | 935.9694 | 3.6821 | 0.0048 | <0.001 |
| 214329_x_at | TNFSF10 | 977.9962 | 4.1102 | 0.0058 | <0.001 |
| 235457_at | MAML2 | 980.6501 | 5.201 | 0.0058 | <0.001 |
| 209908_s_at | TGFB2 | 996.8312 | 5.7026 | 0.006 | <0.001 |
| 210538_s_at | BIRC3 | 1013.8352 | 4.4361 | 0.0062 | <0.001 |
| 1554997_a_at | PTGS2 | 1034.1291 | 5.8764 | 0.0068 | <0.001 |
| 207761_s_at | METTL7A | 1042.1615 | 4.3623 | 0.007 | <0.001 |
| 200953_s_at | CCND2 | 1050.2751 | 5.0309 | 0.0069 | <0.001 |
| 206170_at | ADRB2 | 1057.3049 | 4.9721 | 0.0069 | <0.001 |
| 234973_at | SLC38A5 | 1057.6107 | 5.9017 | 0.0068 | <0.001 |
| 202376_at | SERPINA3 | 1064.3173 | 5.2182 | 0.0069 | <0.001 |
| 214279_s_at | NDRG2 | 1064.576 | 4.9083 | 0.0068 | <0.001 |
| 228575_at | IL20RB | 1085.8307 | 4.4477 | 0.0074 | <0.001 |
| 230030_at | HS6ST2 | 1100.998 | 6.464 | 0.0075 | <0.001 |
| 204470_at | CXCL1 | 1105.2198 | 3.6083 | 0.0075 | <0.001 |
| 208950_s_at | ALDH7A1 | 1105.3772 | 4.8768 | 0.0074 | <0.001 |
| 227070_at | GLT8D2 | 1106.3474 | 3.361 | 0.0073 | <0.001 |
| 238967_at | NULL | 1118.6141 | 5.0828 | 0.0075 | <0.001 |
| 214022_s_at | IFITM1 | 1122.4018 | 5.124 | 0.0075 | <0.001 |
| 223952_x_at | DHRS9 | 1124.3165 | 5.4419 | 0.0075 | <0.001 |
| 1555564_a_at | CFI | 1126.2905 | 4.5651 | 0.0074 | <0.001 |
| 219895_at | FAM70A | 1140.4674 | 4.5329 | 0.0077 | <0.001 |
| 228335_at | CLDN11 | 1153.4728 | 3.4495 | 0.008 | <0.001 |
| 241397_at | NULL | 1156.3402 | 4.5684 | 0.0081 | <0.001 |
| 211122_s_at | CXCL11 | 1182.4334 | 5.5625 | 0.0089 | <0.001 |
| 205625_s_at | CALB1 | 1198.8477 | 5.0276 | 0.0093 | <0.001 |
| 205916_at | S100A7 | 1201.5608 | 4.9949 | 0.0092 | <0.001 |
| 231202_at | ALDH1L2 | 1207.669 | 3.2578 | 0.0092 | <0.001 |

^§^ Gene Symbols are annotated using NetAffx, Affymetrix Analysis Center (www.affymetrix.com/estore/analysis/index.affx). Those labelled ‘NULL’ are either blank, unannotated or have multiple entries. Median gene signal, per sample was used to calculate differential expression. Exon array mapping performed in Annmap. Log2 fold change (low/high) indicates the magnitude difference between classes. Pfp indicates percent false positive taking into account false discovery rate correction. Pvalue is a uncorrected p-value.
